# Relation of Neutrophil Gelatinase-Associated Lipocalin Overexpression to the Resistance to Apoptosis of Tumor B Cells in Chronic Lymphocytic Leukemia

**DOI:** 10.3390/cancers12082124

**Published:** 2020-07-31

**Authors:** Brigitte Bauvois, Elodie Pramil, Ludovic Jondreville, Elise Chapiro, Claire Quiney, Karim Maloum, Santos A. Susin, Florence Nguyen-Khac

**Affiliations:** 1 Cell Death and Drug Resistance in Lymphoproliferative Disorders Team, Centre de Recherche des Cordeliers, Sorbonne Université, Inserm, Université de Paris, F-75006 Paris, France; elodie.pramil@hotmail.fr (E.P.); ludovic.jondreville@gmail.com (L.J.); elise.chapiro@aphp.fr (E.C.); claire.quiney@upmc.fr (C.Q.); santos.susin@crc.jussieu.fr (S.A.S.); florence.nguyen-khac@aphp.fr (F.N.-K.); 2 Biological Hematology Department, Hospital Group Pitié-Salpêtrière, F-75013 Paris, France; karim.maloum@aphp.fr

**Keywords:** apoptosis, CLL, Mcl-1, NGAL, NGAL-R, relapse, remission, STAT3, survival

## Abstract

The resistance to apoptosis of chronic lymphocytic leukemia (CLL) cells partly results from the deregulated production of survival signals from leukemic cells. Despite the development of new therapies in CLL, drug resistance and disease relapse still occur. Recently, neutrophil gelatinase-associated lipocalin (NGAL), a secreted glycoprotein, has been suggested to have a critical role in the biology of tumors. Thus, we investigated the relevance of NGAL in CLL pathogenesis, analyzed the expression of its cellular receptor (NGAL-R) on malignant B cells and tested whether CLL cells are resistant to apoptosis through an autocrine process involving NGAL and NGAL-R. We observed that NGAL concentrations were elevated in the serum of CLL patients at diagnosis. After treatment (and regardless of the therapeutic regimen), serum NGAL levels normalized in CLL patients in remission but not in relapsed patients. In parallel, NGAL and NGAL-R were upregulated in leukemic cells from untreated CLL patients when compared to normal peripheral blood mononuclear cells (PBMCs), and returned to basal levels in PBMCs from patients in remission. Cultured CLL cells released endogenous NGAL. Anti-NGAL-R antibodies enhanced NGAL-R^+^ leukemia cell death. Conversely, recombinant NGAL protected NGAL-R^+^ CLL cells against apoptosis by activating a STAT3/Mcl-1 signaling pathway. Our results suggest that NGAL and NGAL-R, overexpressed in untreated CLL, participate in the deregulation of the apoptotic machinery in CLL cells, and may be potential therapeutic clues for CLL treatment.

## 1. Introduction

Chronic lymphocytic leukemia (CLL) is a very heterogeneous disease characterized by a peripheral accumulation of abnormal CD5^+^ B lymphocytes in the immune system [1]. The leukemic cells (which are mostly quiescent) mainly accumulate because they are unable to develop a cell death program—even though proliferating pools are found in the bone marrow and lymph nodes [1]. This leads to the progressive failure of the patient’s immune and hematopoietic systems [1]. A large number of parameters contribute to apoptosis resistance in CLL B cells, such as intrinsic defects in the programed cell death machinery and the dysregulated production of survival signals by leukemic cells themselves or by the microenvironment [2]. The treatment of CLL remains a challenge in the clinic because ~15–25% of patients either are refractory to today’s front-line therapies or relapse after treatment [3]. Novel signaling inhibitors targeting B cell receptor (BCR)-associated kinases (i.e., Bruton’s tyrosine kinase (BTK) inhibitors such as ibrutinib) have recently been approved in the USA and Europe for relapsed CLL or untreated CLL bearing a *TP53* abnormality [4,5]. Another alternative strategy involves targeting the B-cell lymphoma-2 (Bcl-2) anti-apoptotic protein, which is overexpressed in B-cell malignancies [6]. Venetoclax (a BH3 mimetic that inhibits the survival function of Bcl-2) has been approved for the treatment of relapsed CLL patients including those bearing a 17p deletion [7]. However, some patients still relapse after treatment with ibrutinib or venetoclax, and others even fail to respond [6]. Therefore, novel therapies are needed to overcome resistance to these drugs, and the identification of new therapeutic targets in CLL therapy is of general interest.

Human neutrophil gelatinase-associated lipocalin (NGAL) is a glycosylated protein from the lipocalin family [8]. The lipocalins’ common secondary and tertiary structure corresponds to a single, eight-stranded antiparallel β-barrel around a central pocket that is capable of binding low-molecular weight ligands [8]. NGAL exists as a ~25-kDa monomer, a ~45-kDa homodimer (the most abundant form in healthy subjects), and a 135-kDa disulfide-linked heterodimer bound to the inactive zymogen form of matrix metalloproteinase-9 (proMMP-9) [8,9]. In humans, NGAL is present in most biological fluids and a wide variety of cell types [8,10,11,12]. In normal tissues, NGAL serves to provide protection against bacterial infection and modulate oxidative stress [8,13]. NGAL’s pocket has the ability to capture siderophores (such as bacterial enterochelin and mammalian endogenous catechols) that bind iron with high affinity, causing iron depletion and thus the inhibition of bacterial cell growth [8,14]. There is now evidence to suggest that NGAL may be a marker of disease status in chronic and acute pathological conditions in general and in inflammatory, metabolic, neurologic and cancer diseases in particular [8,10,11,14,15,16,17,18]. The initial functional studies investigated the role of lipocalin-2 (Lcn-2, the murine homolog of human NGAL) in a mouse model However, Lcn-2 exhibits little homology with human NGAL and notably does not contain the unpaired cysteine that can form the NGAL homodimer and the NGAL-proMMP-9 heterodimer in humans [8]. These facts are crucial when analyzing the specific roles attributed to NGAL in humans, which might be distinct from that of Lcn-2 in mice [8,11,14,19,20]. NGAL’s possible roles are being increasingly explored in various cancer models and have unexpectedly shown that NGAL has both beneficial and detrimental effects on cellular processes associated with tumor development (proliferation, survival, migration, and multidrug resistance) [8,10,11]. These activities have been documented in a broad range of human cancer-derived cell lines (which might not reflect primary tumors). Moreover, the NGAL isoforms and receptors involved in functional studies of NGAL have not been characterized; this might explain NGAL’s contrasting effects. A better understanding of the putative causal relationships between NGAL’s functions and the biology of cancers (including leukemias) might help to improve treatment outcomes.

CLL cells from patients with early (Binet stage A) CLL are known to express NGAL [21]. However, there are no data on changes in levels of NGAL during the progression of CLL and following treatment, or on NGAL’s potential contribution to the course of disease. Hence, we decided to assess levels of NGAL in sera from CLL patients as a function of disease severity and treatment. We also sought to determine whether CLL cells co-express NGAL and specific NGAL receptors, which, in turn, may influence the balance between death and survival of CLL B cells.

## 2. Results

### 2.1. Serum NGAL Levels Are Elevated in Untreated Patients with CLL, and Return to Basal Levels When Patients Achieve Remission

**Figure 1:**
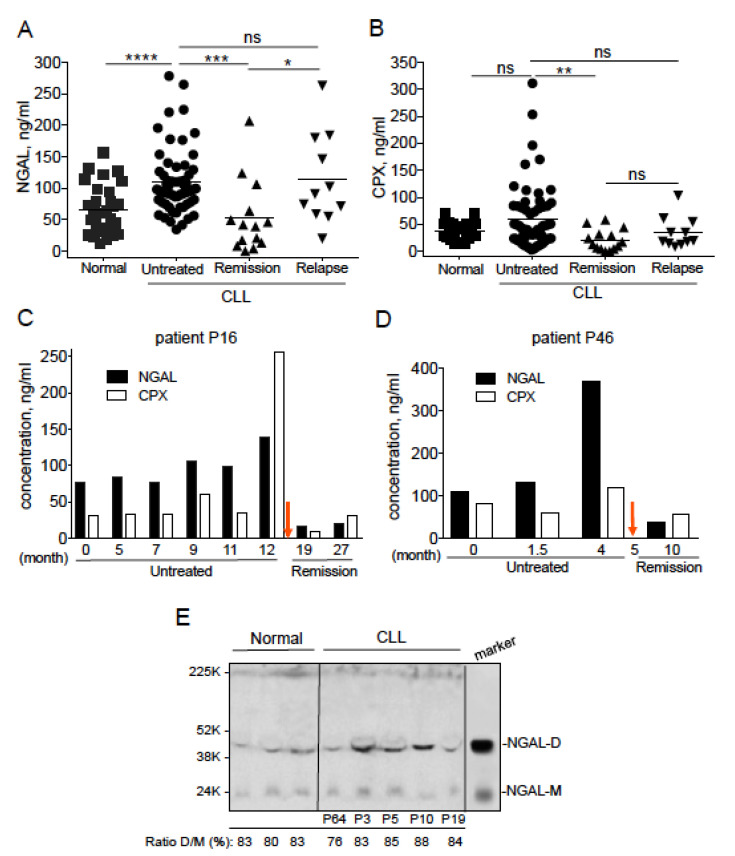
Serum concentrations of neutrophil gelatinase-associated lipocalin (NGAL) in healthy individuals and patients with CLL. ELISAs were performed on 30 normal individuals, 56 untreated CLL patients, 13 CLL patients in remission and 9 CLL relapsed patients. (**A**) NGAL (monomer and dimer), and (**B**) NGAL complexed (CPX) to matrix metalloproteinase-9 (proMMP-9) protein levels were determined. *p* values were calculated using a Mann–Whitney *U*-test; not significant (ns); * *p* < 0.05; ** *p* < 0.01; *** *p* < 0.001 and **** *p* < 0.0001. (**C**,**D**) Levels of NGAL and CPX in two CLL patients (P16 and P46) before treatment (FCR and BR, respectively) or at remission; the red arrow indicates the beginning of the treatment. (**E**) Representative Western blot of NGAL expression in sera of normal and CLL samples (remission P72; untreated P3, P5, P10 and P19); an acute myeloid leukemia (AML) cell lysate was used as a marker of NGAL dimer and monomer. The primary antibody used was anti-NGAL specific for the dimeric and monomeric forms (clone AF1757, goat IgG; R&D Systems). Data are expressed as the ratio (%) between dimer NGAL (NGAL-D) and monomer NGAL (NGAL-M) × 100.

A total of 60 serum samples were obtained from untreated patients with CLL. Forty-eight of the untreated patients were classified as having indolent CLL/stage A (according to the Binet classification), and the remaining 12 were classified as having advanced disease/stage B or C. A total of 25 serum samples were obtained from treated patients with CLL, with 14 patients in remission and 11 in relapse. The patients’ clinical and treatment-related characteristics are summarized in Table 1. For control experiments, we included blood samples from 30 healthy donors. Since NGAL can form a complex with proMMP-9, we measured serum levels of free NGAL and its complex (CPX) at the same time point for a given patient. We found that the serum level of free NGAL was significantly higher in the untreated CLL group (median: 95.94 ng/mL) than in the healthy group (median: 61.82 ng/mL; *p <* 0.0001) (Figure 1A). The elevated NGAL level was not correlated with the Binet stage, *IGHV* status, lymphocyte and neutrophil counts, age, sex, levels of CD38, and genomic aberrations (Table S1). Patients in clinical remission, independently of the therapeutic protocol, elicited a significant reduction in serum NGAL levels (median: 40.51 ng/mL) compared with those of the untreated (median: 95.94 ng/mL; *p* = 0.0001) and relapsed groups (median: 90.7 ng/mL, *p* = 0.012) with a decrease near the level of healthy subjects (median: 61.82 ng/mL; *p* = 0.102) (Figure 1A). Patients in relapse (median: 90.7 ng/mL) showed levels of NGAL higher than those of the healthy group (median: 61.82 ng/mL; *p* = 0.049), and similar to those of the untreated CLL patients (median: 95.94 ng/mL, *p* = 0.727) (Figure 1A). In the remission group, CPX levels (median: 15.21 ng/mL) were significantly lower than in the untreated CLL (median 40.85 ng/mL, *p* = 0.003) and healthy (median 41.84 ng/mL, *p* = 0.0078) groups, and (albeit not significantly) in the relapsed group (*p* = 0.133) (Figure 1B).

We performed a longitudinal analysis of serum samples in 2 CLL patients (P16 and P46) before and after therapy, which confirmed the predictive value of NGAL levels in CLL patients in remission. Patient P16 was treated with fludarabine-cyclophosphamide-rituximab (FCR) 12 months after diagnosis, and was in remission 7 and 15 months after treatment (Figure 1C). Patient P46 was treated with bendamustine and rituximab (BR) 5 months after diagnosis, and was in remission 5 months after treatment (Figure 1D). Before treatment, lymphocyte counts were elevated and increased with time; after treatment, the lymphocyte levels of both patients fell to normal values (i.e., those observed in healthy controls; 1–4 G/L) (Table 2).

In parallel, before treatment, the patients’ serum levels of NGAL and CPX had increased over time (Figure 1C,D). After treatment, the serum levels of both markers fell to normal or below-normal values (Figure 1C,D). The serum levels of NGAL and CPX correlated at least with the lymphocyte counts of patient P16 (Table 2). Hence, the reduction in serum NGAL levels appears to be associated with a good treatment response (regardless of the therapeutic regimen).

Representative immunoblotting with an antibody that bound to both dimeric (45 kDa) and monomeric NGAL (25 kDa) revealed the presence of endogenous NGAL (mainly as a dimer: ≥80%) in serum samples from healthy controls and from patients with CLL (four untreated patients and one patient in remission) (Figure 1E).

### 2.2. The Expression of NGAL Reveals Differences between PBMCs from Normal Controls and Patients with CLL

**Figure 2:**
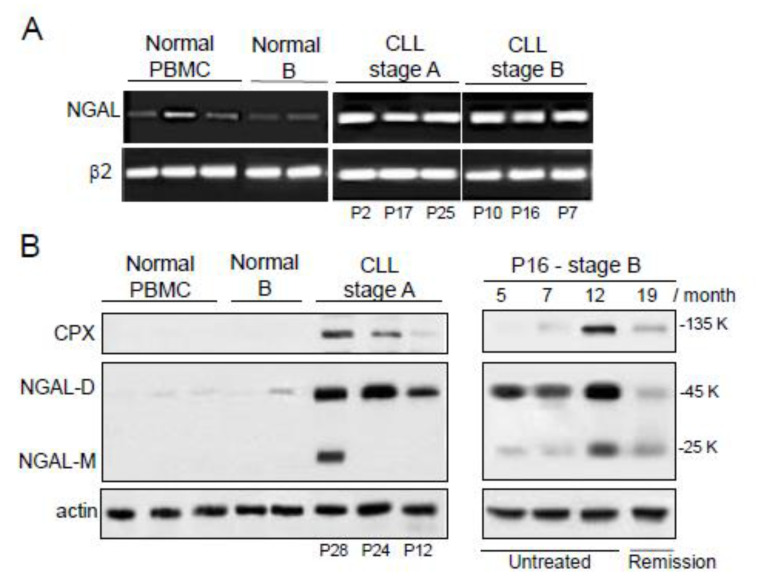
Expression profiles of NGAL in peripheral blood mononuclear cells (PBMCs) from healthy individuals, untreated CLL patients and patient P16 before FCR treatment or at remission. (**A**) Representative RT-PCR results for three PBMCs and two purified CD19^+^ B cells from healthy donors, and six CLL cells from untreated patients. (**B**) Representative Western blot (non-reducing conditions) results (left panel) for three PBMCs and two purified CD19^+^ B cells from healthy donors, and three CLL cell samples from untreated CLL patients (stage A). Western blot (non-reducing conditions) results (right panel) for CLL cells of patient P16 (stage B) before FCR treatment (months 5, 7, and 12) or at remission (month 19). The primary antibodies used were anti-NGAL specific for the dimeric and monomeric forms (R&D Systems) and anti-MMP-9 which recognizes CPX (Abcam).

Earlier studies showed that CLL cells from Binet stage A patients express NGAL protein and its complex [21,22]. Here, we completed these data by analyzing the expression of NGAL in CLL cells compared to normal peripheral blood mononuclear cells (PBMCs) and normal B cells. As exemplified in Figure 2A, NGAL transcripts were strongly expressed in CLL cells, in contrast to normal PBMCs and B cells. Transcript levels of NGAL did not appear to be related to disease progression (Figure 2A). Accordingly, normal PBMCs and B cells were found to express undetectable to very low baseline levels of NGAL protein while untreated CLL cells expressed elevated levels of NGAL (mainly as a dimer) (Figure 2B, left panel). The levels of CPX were detectable only in CLL cells (Figure 2B). When normalized against actin levels, the differences in NGAL protein expression between CLL cells, normal PBMCs and normal B cells were statistically significant (i.e., normal (n = 7) vs. CLL (n = 11) cells; *p <* 0.001). There were no differences between Binet stage A patients and stage B/C patients in *NGAL* protein levels (i.e., A (n = 6) vs. B/C (n = 5); *p* = 0.181). In addition, the release of NGAL by primary CLL cells in vitro and under basal conditions, was studied in 33 CLL cases: six samples were strongly positive (>10 ng/mL for 10^6^ cells), 16 displayed an intermediate signal (0.3–3.7 ng/mL for 10^6^ cells) and the last 11 were negative (<0.1 ng/mL for 10^6^ cells) (Figure S1). The levels of CPX released by CLL cells were undetectable in all samples tested (<0.1 ng/mL).

Furthermore, the levels of NGAL protein expression could be measured in CLL cells from patient P16 (stage B) before treatment and at remission (Figure 2B, right panel). In line with the results for serum samples (Figure 1C), CLL cells from this patient displayed high protein levels of NGAL 5 and 7 months after diagnosis, and these levels were even higher 12 months after diagnosis; after remission (month 19 corresponding to 7 months after FCR treatment), most of P16’s blood cells recovered as normal PBMCs and consistently the levels of NGAL fell to decrease near the level of controls (Figure 2B, right panel).

### 2.3. NGAL-R Is Overexpressed in CLL Cells from Untreated Patients, and Downregulated in CLL Cells from Patients in Remission

**Figure 3:**
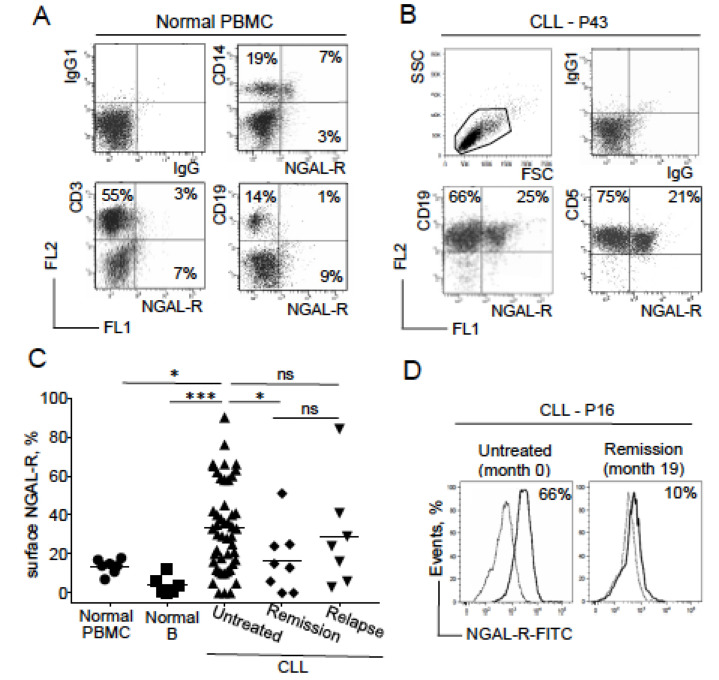

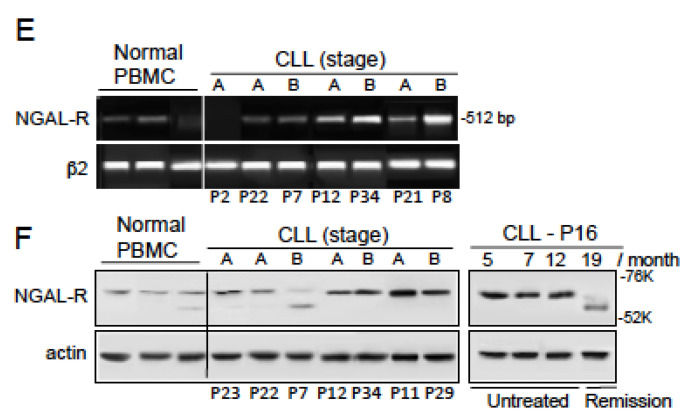
Expression profiles of NGAL receptor (NGAL-R) in PBMCs from healthy individuals, and CLL cells from patients before and after therapeutic treatment. (**A**) Representative cytograms of normal PBMCs stained with rabbit IgG-FITC/mIgG1-PE, NGAL-R-FITC/CD14-PE, NGAL-R-FITC/CD3-PE, NGAL-R-FITC/CD19-PE Abs. (**B**) Representative cytogram of CLL cells from one untreated patient, stained with rabbit IgG-FITC/mIgG1-PE, NGAL-R-FITC/CD19-PE and NGAL-R-FITC/CD5-PE Abs. (**C**) NGAL-R surface levels were determined, (with rabbit IgG-FITC and NGAL-R-FITC) in PBMCs and purified CD19^+^ B cells from healthy individuals, and CLL cells from patients before treatment or at remission or relapse. The data are presented as mean ± SEM (normal PBMCs, n = 7 healthy donors; isolated B cells, n = 6 healthy donors; CLL cells, n = 47 untreated patients, n = 8 patients in remission and n = 7 relapsed patients). *p* values were calculated using a Mann–Whitney *U*-test; not significant (ns); * *p* < 0.05; *** *p* < 0.001. (**D**) Expression of surface NGAL-R in CLL cells from patient P16 before treatment and at remission; cytogram of CLL cells stained with rabbit IgG-FITC (broken line) or NGAL-R-FITC Abs at diagnostic (month 0) and at remission at month 19 (month 19). (**E**) Representative RT-PCR results for NGAL-R in PBMCs from three healthy individuals and CLL cells from seven untreated patients. (**F**) Representative Western blot (reducing conditions) results for NGAL-R protein in PBMCs from three healthy individuals, CLL cells from seven untreated patients, and CLL cells of patient P16 before treatment (month 5, 7, and 12) and at remission (month 19).

A specific membrane-bound receptor for NGAL (also known as solute carrier family 22, member 17 (SLC22A17), and referred to as NGAL-R) has been identified in human epithelial cells [23]. Using a FITC-conjugated anti-SLC22A17, we show that only a very small proportion of normal PBMCs expressed NGAL-R (Figure 3A). The receptor was expressed by some CD14^+^ cells (a monocyte lineage) but not by CD19^+^ (B cell lineage) or CD3^+^ (T cell lineage) cells (Figure 3A). In marked contrast, CD5^+^CD19^+^ B cells from CLL patients expressed NGAL-R (Figure 3B). Surface expression of NGAL-R was significantly higher in the majority of leukemic cell samples from the untreated CLL group (n = 47) than in normal PBMCs and B cells (2.4 and 8.4 times more, respectively; *p* = 0.016 and *p* = 0.0003, respectively; Figure 3C). Surface expression of NGAL-R by CLL cells from patients in remission (regardless of the therapeutic regimen) was significantly lower than in the untreated CLL group (*p* = 0.040) (Figure 3C), and was similar to those in normal PBMCs (*p* = 0.975). In relapsed CLL patients, NGAL-R levels were similar to that observed in the untreated group, but higher (albeit not significantly) than in the remission group (*p* = 0.320). Surface NGAL-R expression levels could be quantified for patient P16′s CLL cells before FCR treatment and at remission; the surface NGAL-R level was high before treatment but fell after treatment with FCR (Figure 3D). The level of surface NGAL-R expression in CLL cells from untreated patients appeared to be independent of clinical features [24].

Representative RT-PCR and Western blot experiments confirmed that most CLL cell samples expressed the SLC22A17 transcript (512 bp) and the SLC22A17A protein (60 kDa) at higher levels than normal PBMCs did (Figure 3E,F). A distinct NGAL-R isoform with a lower size could be detected in two samples of normal PBMCs and CLL cells (Figure 3F) which might correspond to a less glycosylated protein [25]. Western blotting experiments showed that NGAL-R was clearly present in patient P16′s CLL cells before treatment but only weakly present in P16’s PBMCs after remission (Figure 3F). Taken as a whole, these data indicate that (as seen for NGAL) the expression of NGAL-R in leukemic B cells is a marker of CLL disease but not of its severity. Furthermore, the downregulation of NGAL-R expression appears to be associated with a good treatment response.

### 2.4. Blocking NGAL-R Induces Death in NGAL-R^+^ CLL Cells

**Figure 4:**
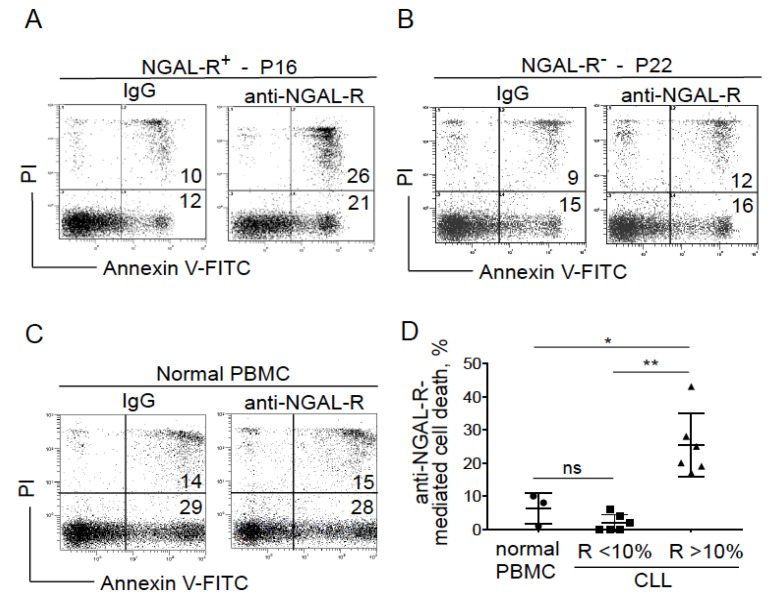
Anti-NGAL-R antibodies induce the death in CLL cells. (**A**–**C**) Representative cytograms of (**A**) NGAL-R^+^ CLL cells (44%; P16), (**B**) NGAL-R^−^ CLL cells (0%; P22) and (**C**) normal PBMCs cultured for 24 h in the presence or absence of 20 μg/mL anti-NGAL-R antibodies or rabbit IgG (isotype control); detection of apoptotic cells after annexin-V-FITC/PI staining and flow cytometry. Results are as log PI fluorescence intensity (*y*-axis) vs. log annexin-V-FITC fluorescence intensity (*x*-axis). The percentage of annexin-V-positive cells is shown. (**D**) Quantification of anti-NGAL-R-mediated survival levels in PBMCs from healthy individuals and CLL cells from untreated patients. The percentage of anti-NGAL-R-mediated survival is determined by subtracting the percentage of annexin-positive cells in the presence of rabbit IgG from the percentage of annexin-positive cells in the presence of anti-NGAL-R Ab. The data are presented as mean ± SD (normal PBMCs, n = 3; CLL cells, n = 12, with n = 6 NGAL-R^+^ (≥10%) and n = 6 NGAL-R^−^ (<10%); Statistical relevance was assessed with the unpaired *t*-test; not significant (ns); * *p* < 0.05; ** *p* < 0.01.

We studied whether neutralizing antibodies against NGAL-R influenced the spontaneous death of cultured CLL cells. For this purpose, we examined the effects of anti-NGAL-R antibodies and control rabbit IgG (20 μg/mL, for 24 h) on the viability of CLL cells obtained from untreated patients. As exemplified in Figure 4A, a lethal effect was observed for NGAL-R^+^ CLL cells after their incubation with the anti-NGAL-R antibodies compared with control (IgG) experiments (i.e., annexin V^+^ cells). In contrast, no effect was observed in NGAL-R^−^ CLL cells treated either with the anti-NGAL-R or the isotype (i.e., annexin V^+^ cells; Figure 4B). Similarly, normal PBMCs (i.e., cells that express low levels of NGAL-R) were not affected by the addition of anti-NGAL-R antibodies (Figure 4C). As summarized in Figure 4D, anti-NGAL-R antibodies significantly increased cell death in the NGAL-R^+^ CLL group (mean: 25.3% ± SD: 9.6) compared to normal PBMCs (6.3% ± 4.7) and NGAL-R^low^ CLL cells (2% ± 2.5). These data strongly suggest that neutralizing anti-NGAL-R antibodies induce CLL cell death—perhaps by blocking the interaction between endogenous NGAL and its surface receptor.

### 2.5. NGAL Protects CLL Cells from Spontaneous Death

**Figure 5:**
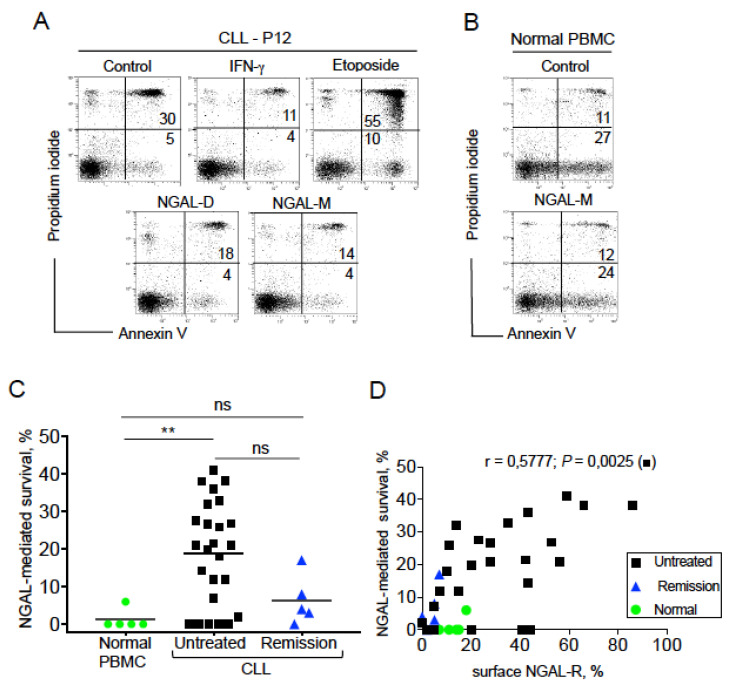
NGAL protects CLL cells from spontaneous death. (**A**) Representative cytograms of CLL cells cultured for 24 h in the presence or absence of 100 nM recombinant human NGAL (dimers and monomers), 1000 U/mL IFN-γ or 1 μM etoposide; detection of apoptotic cells after annexin-V-FITC/PI staining and flow cytometry. The percentage of annexin-V-positive cells is shown. (**B**) Representative cytograms of PBMCs from one healthy donor cultured for 24 h in the presence or absence of 100 nM NGAL monomers, and cell death was assessed as described in (**A**). (**C**) NGAL-mediated survival levels were determined in PBMCs from healthy individuals and CLL cells from patients before treatment or at remission. The percentage of NGAL-mediated survival is determined by subtracting the percentage of annexin-positive cells in the absence of NGAL from the percentage of annexin-positive cells in the presence of NGAL, and divided by the percentage of annexin-positive cells in the absence of stimulus x 100. The data are presented as mean ± SEM (normal PBMCs, n = 5; CLL cells from untreated patients, n = 25; CLL cells from patients in remission, n = 5). *p* values were calculated using a Mann–Whitney U-test; not significant (ns); ** *p* < 0.01. (**D**) Correlation between the levels of surface NGAL-R and NGAL-mediated survival in normal PBMCs (n = 5), CLL cells from untreated patients (n = 25) and PBMCs from patients in remission (n = 5); Spearman’s correlation coefficient (r) and the *p*-value are shown for untreated CLL.

We next looked at whether NGAL influenced the balance between death and survival in CLL cells obtained from untreated patients. To this end, we used two commercially available recombinant human NGAL proteins: a dimer (hereafter referred to as NGAL-D) and a monomer NGAL (hereafter referred to as NGAL-M) (Figure S2). Firstly, we assessed the effects of the two NGALs (100 nM, for 24 h) on the viability of NGAL-R^+^ CLL cells. Etoposide (1 μM) was used as a positive control for CLL cell death [26], and recombinant interferon (IFN)-γ (1000 U/mL) was used as a positive control for CLL cell survival [27]. As expected, etoposide treatment resulted in a high level of cell death (i.e., annexin V^+^ cells; Figure 5A). In contrast, the proportion of annexin V^+^ cells was lower after treatment with NGAL-D, NGAL-M or IFN-γ than in control (untreated) experiments (Figure 5A). As shown in Figure 5B, PBMCs from healthy subjects (i.e., cells that express low levels of NGAL-R) were not affected by the addition of 100 nM NGAL. The NGAL proteins had a dose-dependent effect on the survival of CLL cells [28], and a dose of 100 nM NGAL was investigated in all subsequent experiments. In CLL cells from untreated patients, NGAL induced cell survival in 19 of the 25 CLL samples tested (Figure 5C), and this protective effect appeared independent of the Binet stage (stage A (n = 15) vs. stage B/C (n = 4), *p* = 0.883). In contrast, NGAL did not affect the viability of PBMCs from healthy subjects (Figure 5C). The survival rates were lower (albeit not significantly) in the remission group than in the untreated group (*p* = 0.086). We found a positive correlation between the NGAL-R expression and NGAL-mediated CLL cell survival for the untreated group (*p* = 0.0025). Hence, these data indicate that NGAL protects CLL cells from death, and that NGAL’s survival-promoting effect is specific for NGAL-R^+^ CLL cells.

### 2.6. NGAL Counteracts the Intrinsic Apoptosis Pathway in CLL Cells

**Figure 6:**
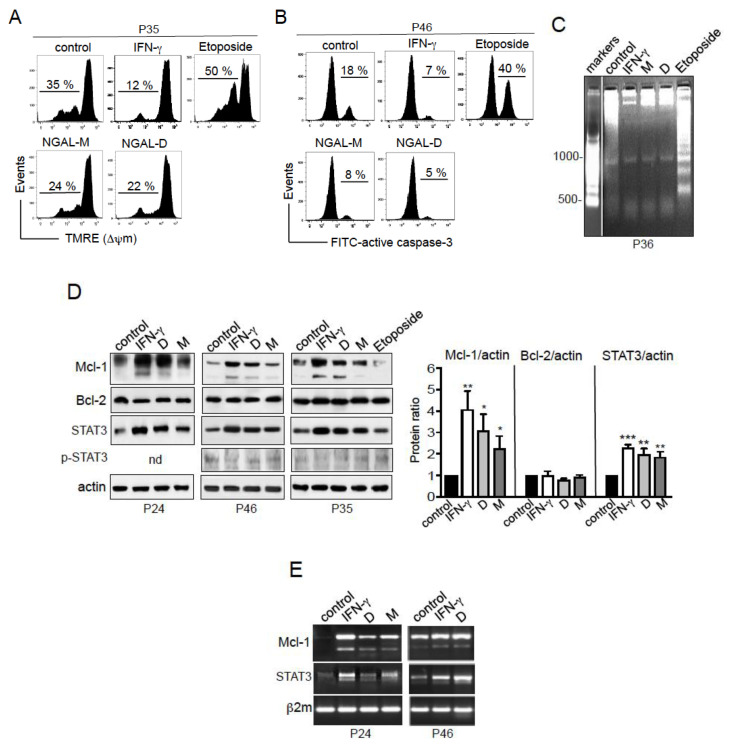
NGAL upregulates Mcl-1 and STAT3 in CLL cells. (**A**) Representative cytograms of CLL cells cultured for 24 h in the presence or absence of NGAL dimers or monomers (100 nM), IFN-γ (1000 U/mL) or etoposide (1 μM); cells were labelled with the FL2 probe TMRE. The percentages refer to ΔΨ_m_ loss. (**B**) Representative cytograms of CLL cells cultured for 24 h in the presence or absence of NGAL dimers or monomers (100 nM), IFN-γ (1000 U/mL) or etoposide (1 μM); cells were stained with rabbit IgG-FITC or anti-active caspase-3-FITC and then examined by flow cytometry. (**C**) CLL cells cultured for 30 h in the presence or absence of NGAL dimers or monomers (100 nM), IFN-γ (1000 U/mL) or etoposide (1 μM); DNA fragmentation was evaluated by the detection of an oligonucleosome ladder by agarose gel electrophoresis. (**D**) CLL cells were cultured for 24 h in the presence or absence of NGAL dimers or monomers (100 nM), IFN-γ (1000 U/mL) or etoposide (1 μM); after which lysates were Western blotted (reducing conditions) with antibodies against Bcl-2, Mcl-1 (Mcl-1_L_ and Mcl-1_S_), STAT3, p^Y705^-STAT3 and actin. Three representative experiments (n = 6 in all) are shown. Data (n = 6) are expressed as the ratio between the analyte proteins and actin, and presented as mean ± SEM. Statistical relevance was assessed with the unpaired *t*-test; not done (nd); * *p* < 0.05; ** *p* < 0.01; *** *p* < 0.001. (**E**) CLL cells were cultured for 18 h in the presence or absence of NGAL dimers or monomers (100 nM) or IFN-γ (1000 U/mL). Then, the cDNAs were used as templates for PCR reactions using specific primers for Mcl-1 (Mcl-1_L_ and Mcl-1_S_), STAT3 and β2-microglobulin.

In further experiments, we investigated the molecular mechanisms underlying NGAL’s ability to protect CLL cells from cell death. The mitochondrial (intrinsic) pathway controls the balance between apoptosis and survival in CLL cells [29,30]. Etoposide activates the intrinsic apoptotic pathway, with the disruption of mitochondrial transmembrane potential (ΔΨ_m_), caspase activation, and DNA fragmentation [31,32]. In contrast, IFN-γ protects CLL cells from apoptotic death [27]. Here, the exposure of cells to NGAL (D or M; 100 nM) or IFN-γ (1000 U/mL) for 24 h prevented ΔΨ_m_ loss (Figure 6A). As expected, etoposide (1 μM) treatment resulted in the increased dissipation of the ΔΨ_m_ (Figure 6A). Furthermore, we assessed the level of expression of active caspase-3 involved in mitochondrial apoptosis [33]. In contrast to etoposide’s triggering of caspase-3 activation, CLL cells treated with NGAL or IFN-γ displayed lower levels of active caspase-3 than untreated cells (Figure 6B). Nuclear DNA fragmentation (which is mediated by caspase-activated DNAse [31]) was observed in etoposide-treated CLL cells (Figure 6C). In contrast, DNA fragmentation was lower in NGAL- and IFN-γ-treated CLL cells than in untreated cells (Figure 6C). These data collectively indicate that NGAL dimers and monomers prevent spontaneous apoptosis in cultured CLL cells.

### 2.7. NGAL Upregulates the Expression of Mcl-1 and STAT3 in CLL Cells

The increase in ΔΨ_m_ is mainly dependent on the action of the anti-apoptotic proteins Bcl-2 and Mcl-1 belonging to the Bcl-2 family [33]_._ Both Bcl-2 and Mcl-1 proteins are constitutively expressed in CLL cells, and are involved in the cells’ ability to avoid apoptosis [30]. We analyzed the protein levels of Mcl-1 and Bcl-2 in CLL cells in the absence or presence of stimuli (NGAL D, NGAL M, IFN-γ or etoposide. At 24 h of culture, untreated cells expressed detectable levels of Mcl-1 and Bcl-2 (Figure 6D left panel, 3 separate experiments). Treatment with NGAL or IFN-γ led to significantly greater Mcl-1 levels (relative to untreated cells), whereas no significant differences were observed for levels of Bcl-2 (Figure 6D, left panel). Notably, the shorter isoform Mcl-1 was weakly induced in CLL cells stimulated with IFN-γ or NGAL-D (Figure 6D). In line with earlier studies [34], Mcl-1 levels were lower in etoposide-treated cells than in untreated cells (Figure 6D, left panel). When normalized against actin levels, the Mann–Whitney test confirmed the significant upregulation of Mcl-1 protein in NGAL- or IFN-γ-treated CLL cells (Figure 6D, right panel; n = 6 separate experiments). In parallel, RT-PCR assays after 18 h of culture showed that Mcl-1 transcripts encoding the Mcl-1 protein were upregulated in NGAL- and IFN-γ-treated CLL cells, when compared with untreated cells (Figure 6E). Taken as a whole, these results strongly suggest that NGAL counteracts mitochondrial-dependent CLL cell death by upregulating the transcription of the survival protein Mcl-1.

When overexpressed or activated by various stimuli (including IFN-γ), the signal transducer and activator of transcription (STAT) member STAT3 can activate target genes including *STAT3* itself and the gene coding for Mcl-1 (*MCL1*) [35,36]. The above-described upregulation of *MCL1* transcription in CLL cells by NGAL or IFN-γ (Figure 6E) was indeed accompanied by a concomitant elevation in *STAT3* transcription (Figure 6E). The activation of STAT3 through Tyr-705 phosphorylation is known to be transient in CLL cells (from 5 min to 15 h) [37], which can explain that the relative levels of p^Y705^-STAT3 in CLL cells at 24 h of culture were found to be similar in NGAL- and IFN-γ-treated cells and in untreated cells (Figure 6D). However, the significant elevation at 24 h in total STAT3 levels in NGAL- and IFN-γ-treated cells indicates that STAT3 was being activated through Tyr-phosphorylation (Figure 6D). As seen for Mcl-1, the Mann–Whitney test confirmed the significant upregulation of STAT3 in NGAL- or IFN-γ-treated CLL cells (Figure 6D, right panel; n = 6 separate experiments).

### 2.8. NGAL-Mediated CLL Cell Survival Involves the Src/STAT3/Mcl-1 Signaling Pathway

**Figure 7:**
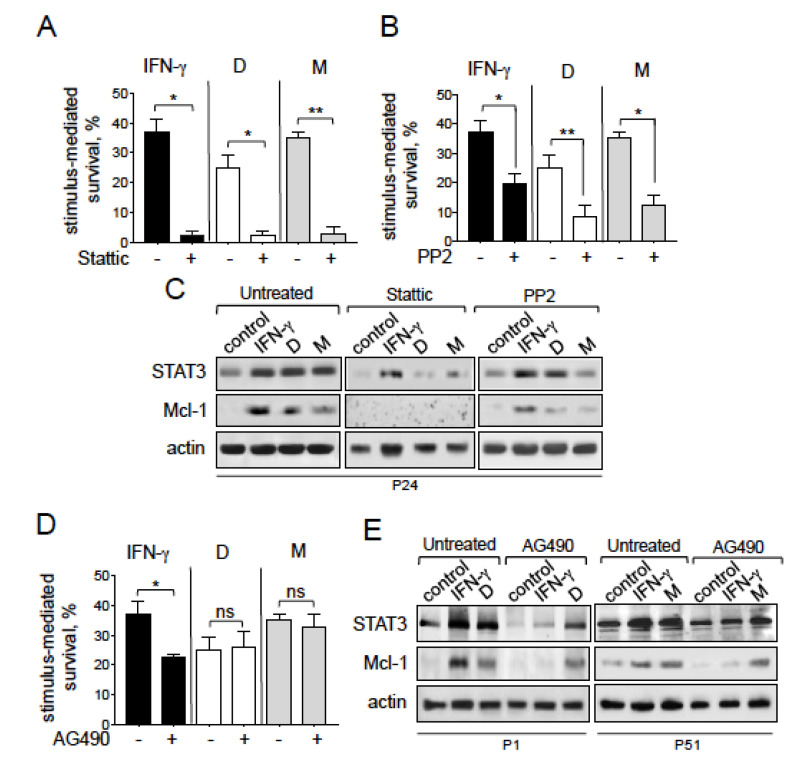
Exogenous NGAL induces CLL cell survival through the Src/STAT3/Mcl-1 signaling pathways. (**A**–**C**) CLL cells were cultured for 24 h in the presence or absence of NGAL dimers or monomers (100 nM) or IFN-γ (1000 U/mL) after a 15 min pretreatment with (**A**) 5 μM STAT3 inhibitor, (**B**) 10 μM PP2 (an Src family kinase inhibitor), or (**D**) 10 μM AG490 (a JAK2 inhibitor); after which NGAL-mediated survival was assessed as described in Figure 4. The data are presented as mean ± SEM (n = 3). Statistical relevance was assessed with the paired *t*-test; not significant (ns); * *p* < 0.05; ** *p* < 0.01. (**C**,**E**) CLL cells were cultured for 24 h in the presence or absence of NGAL dimers or monomers (100 nM) or IFN-γ (1000 U/mL) after a 15 min pretreatment with (**C**) 5μM STAT3 inhibitor, 10 μM PP2, or (**E**) 10 μM AG490; after which lysates were Western blotted (reducing conditions) with antibodies against STAT3, Mcl-1 and actin. Representative experiments (n = 3) are shown.

We next investigated whether the STAT3/Mcl-1 pathway was involved in the NGAL-mediated survival of CLL cells. In general, STAT3 can be activated by cytoplasmic tyrosine kinases, including the JAK2 and Src kinases [35,37,38,39]. In CLL, the JAK2/STAT3 pathway is constitutively activated and its inhibition leads to CLL cell death [40]. To this end, we treated cells with the following pharmacological inhibitors: Stattic, a selective STAT3 activation inhibitor that blocks the phosphorylation of STAT3 (on tyr-705) and therefore prevents it from binding to upstream kinases [41]; PP2, a selective Src family kinase inhibitor [42] and AG490, a selective JAK2 inhibitor [43]. For each pharmacological inhibitor, we applied the highest concentration that did not markedly affect the viability of CLL cells (i.e., a cell death rate of no more than 20%, relative to basal levels). As shown in Figure 7A, treatment with Stattic (5 μM) almost totally inhibited the survival of CLL cells mediated by NGAL (D and M) or IFN-γ. This inhibition was associated with the concomitant downregulation of STAT3 and Mcl-1 proteins (Figure 7C), indicating that the STAT3/Mcl-1 pathway was involved in the survival of CLL cells mediated by NGAL or IFN-γ. Furthermore, NGAL-mediated CLL cell survival was significantly prevented by PP2 (10 μM) (Figure 7B) but not by AG490 (10 μM) (Figure 7D); accordingly, the relative levels of STAT3 and Mcl-1 proteins were downregulated in the presence of PP2 (Figure 7C) but remained almost unchanged in the presence of AG490 (Figure 7E). As expected, AG490 markedly blocked IFN-γ-mediated CLL cell survival (Figure 7D) by downregulating the protein levels of STAT3 and Mcl-1 (Figure 7E). Taken as a whole, these results indicate that NGAL-mediated CLL cell survival is dependent on the Src/STAT3/Mcl-1 pathway.

## 3. Discussion

Oncologists and cancer biologists are now focusing on NGAL’s potential as an early diagnostic marker, a prognostic marker, and an indicator of treatment effectiveness for a number of solid tumors [44,45]. To the best of our knowledge, the potential value of NGAL in the pathogenesis of CLL has not previously been investigated. The present study provides evidence that NGAL may have a diagnostic value in untreated CLL and a predictive value in patients in remission; in addition, our findings strongly suggest the involvement of NGAL in the resistance to apoptosis of neoplastic CLL cells through an autocrine process.

Serum levels of NGAL (mainly present as dimers) were significantly higher in untreated CLL patients than in healthy controls. After treatment, and independently of the treatment regimen applied, these serum levels normalized in patients in remission. In peculiar, a longitudinal study of two CLL patients who achieved clinical remission after treatment with FCR or BR confirmed the results obtained in the small cohort of patients in remission: serum NGAL levels were much lower after treatment than at the full-blown disease stage, and returned to control values. The enhanced NGAL levels observed in serum might be explained as the expression of NGAL release from circulating CLL lymphocytes. Indeed, the blood concentrations of NGAL are known to be influenced by changes in leukocyte profiles as well as by the activation state of cells [46]. Accordingly, our data clearly show that peripheral blood CLL cells (in contrast to normal PBMCs and normal B cells), constitutively expressed NGAL; the NF-κB signaling pathway, activated in most cancers including CLL, regulates the transcription of NGAL [8], and this likely explains the enhanced levels of NGAL in CLL cells. Moreover, the natural forms of NGAL molecules in CLL cells are identical to those present in serum, and both cellular and serum levels concomitantly fall near to normal basal values following clinical remission of patients. Finally, our longitudinal study showed a significant correlation between serum levels of NGAL and lymphocyte count in one CLL patient. Taken as a whole, these observations strongly suggest that serum NGAL levels detected in untreated CLL patients are derived from circulating CLL lymphocytes.

A putative functional role for NGAL in CLL has not yet been reported. Early studies showed enhanced serum levels of TNF-α, VEGF and proMMP-9 in patients with CLL [47,48,49], in correlation with their overexpression and release by CLL lymphocytes [49,50,51] and their implication in CLL cell survival [37,50,52,53]. NF-κB also regulates the transcription of these inflammatory molecules [54,55]. Hence, we wanted to find out if the aberrant expression of NGAL might contribute to CLL pathogenesis. To this end, we first investigated the presence of specific receptors for NGAL in CLL cells. The present study is the first to have shown that the receptor for NGAL (SLC22A17; absent or weakly expressed in normal PBMCs) is strongly expressed by tumoral B cells from untreated CLL patients. Both NGAL and NGAL-R are upregulated in CLL, a situation similar to their expression pattern in various solid tumors where they are associated with clinical prognosis [56,57,58,59,60]. After treatment, total and surface NGAL-R levels fell to baseline levels in patients achieving clinical remission. Therefore, the modulation of NGAL and NGAL-R in CLL suggests that they might be of clinical value in newly diagnosed CLL patients and those in remission.

How, then, can NGAL-R expression be increased in CLL? The Runx family of transcriptional regulators activates or represses gene expression [61]. In murine bone marrow cells, NGAL-R expression is regulated by Runx3 as an activator and Runx1 as a repressor [62]. Runx3 is expressed in human cells of hematopoietic origin [63] and increased in most cell lines of leukemia [61,64]. An elevated Runx3 expression is significantly correlated with poor overall survival in patients with leukemia, which seems consistent with the role of Runx3 as an oncogene [61]. It remains to be seen whether NGAL upregulation depends on Runx3 transcriptional activity in CLL.

The initial study performed by Lagneaux et al. [65] showed that adhesion of CLL cells to bone marrow stromal cells rescued them from apoptosis and extended their life span in vitro. There is now evidence that in CLL, the microenvironment plays a critical role in promoting tumor cell recruitment, activation, survival and expansion [66,67,68,69]. In lymphoid organs, circulating CLL cells are surrounded by a supportive microenvironment including antigens (e.g., CD40L, B-cell receptor/BCR, VCAM-1, CD49d), cytokines (e.g., IL-6, IL-10) and chemokines (e.g., CXCL12, CXCL13) and extracellular matrix proteins (e.g., fibronectin) provided by other CLL cells, T and NK cells, monocyte-derived nurse-like cells and stromal cells, all of which provide additional survival and anti-apoptotic signals to CLL cells [2,66,67,68,69,70,71]. Whether the tumor microenvironment upregulates the expression of NGAL and NGAL-R in CLL cells deserves investigation. Thus, it is possible here that the high levels of NGAL detected in vivo (serum) and in vitro (culture media) are related to specific malignant B cell clones sensitive to the CLL microenvironment. Additional investigations are needed to address the participation of the tumor microenvironment in NGAL regulation in CLL.

Furthermore, we showed that both forms of NGAL (dimers and monomers) promote the survival of primary NGAL-R^+^ CLL cells by blocking cell apoptosis through the intrinsic pathway. The anti-apoptotic Mcl-1 protein is a critical regulator of the intrinsic pathway [72]. Resistance to the apoptosis of CLL B cells partly results from the high expression of Mcl-1, which correlates with a poor prognosis and chemotherapy resistance [73,74]. The Mcl-1 promoter is a STAT3 target [36,72]. Previous studies showed that the increase in CLL viability induced by sustained BCR engagement (using anti-IgM antibodies or B-cell activating factor/BAFF) is accompanied by STAT3 activation and Mcl-1 upregulation [75,76,77]. Moreover, once proMMP-9 has bound to its docking receptors α4β1 integrin and CD44, STAT3 activation is required for Mcl-1 mediated CLL cell survival [37]. The results of our experiments with a specific STAT3 activation inhibitor indicate that NGAL-mediated CLL cell survival involves the STAT3/Mcl-1 pathway. Several tyrosine kinases are known to activate STAT3, including JAK2 (usually activated by IFN-γ) [78], the Src family members Lyn, Fyn and c-Src [37,78,79,80] and the SYK family tyrosine kinase Syk [77,81]. In CLL cells, STAT3 is either activated by Lyn [37], Syk [77,81] or JAK2 [40,76]. Here, we provide evidence that the sequence of events leading to NGAL-mediated CLL cell survival can be attributed, at least, to the likely activation of an Src family kinase, which activates STAT3, which, in turn, upregulates Mcl-1. With regard to Src family members, Lyn is known to be overexpressed in CLL cells, and contributes to their survival [82,83]. The inhibitor PP2 blocks Lyn activity in primary CLL cells [82]. It remains to be seen whether NGAL’s effect on survival depends on Lyn activation. Importantly, Src kinases (including Lyn and c-Src) can activate BTK [84]. One study suggested that STAT3 was involved as a BTK substrate [85]; we therefore wondered whether NGAL-mediated STAT3 activation first activated Src and then BTK - leading to further STAT3 activation. However, our results showed that the BTK inhibitor ibrutinib affects neither NGAL-mediated CLL cell survival nor the NGAL-R expression level (Figure S3)—indicating that BTK is not involved in NGAL signaling. Lastly, recent work has demonstrated that recombinant human NGAL (Sigma) mediates sunitinib resistance in renal tumor cells by inducing STAT1 activation [86]. In our experiments, the fact that STAT1 protein was not activated by NGAL (Figure S4) rules out STAT1′s involvement in NGAL-mediated survival signaling.

**Figure 8:**
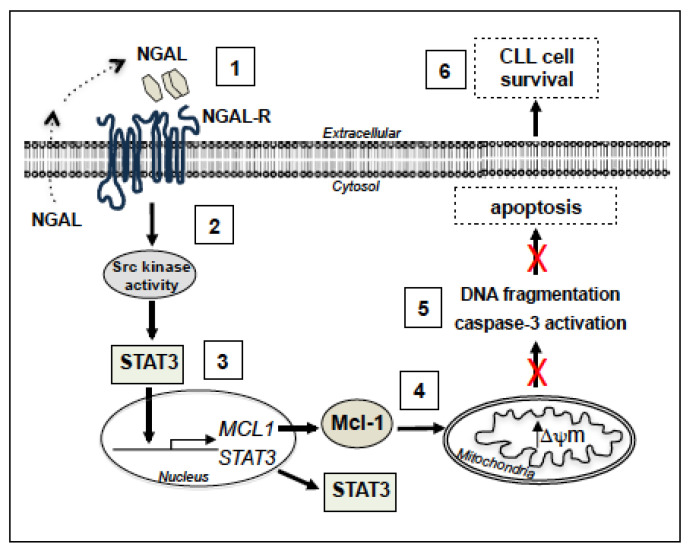
Putative model for the involvement of cell signaling pathways in the induction of survival by NGAL in CLL cells. By binding to surface NGAL-R (**1**), NGAL (dimers and monomers) likely leads to the activation of an Src family kinase (**2**), which, in turn, activates STAT3. STAT3 dimer enters the nucleus and binds the promoters of *STAT3* and *MCL1* (**3**). Following *STAT3* and *MCL1* transcription, STAT3 and Mcl-1 proteins accumulate in the cytoplasm, and Mcl-1 exerts its anti-apoptotic activity by preventing mitochondrial depolarization (**4**), leading to the inhibition of caspase-3 activation and DNA fragmentation (**5**), ultimately favoring cell resistance to apoptosis (**6**). The dotted arrow suggests an autocrine NGAL stimulation loop for CLL cell survival.

It is not clear how NGAL-R activates an Src family kinase. NGAL-R belongs to the SLC22 family of organic ion transporters [25]. This type of transmembrane protein consists of twelve transmembrane (TM) helical segments, organized in two bundles of six TMs, connected by a large loop [25]. One can hypothesize that NGAL-R binds to, and cooperates with, other surface receptors that are themselves capable of inducing survival in CLL cells. Candidate receptors include β1 integrin, CD44, and CD38. Indeed, the binding of proMMP-9 to α4β1 integrin and CD44 induced an intracellular signaling pathway (consisting of Lyn kinase activation, STAT3 phosphorylation, and Mcl-1 activation) that promoted the survival of CLL cells [37]. CD38 exerts a prosurvival function in CLL cells by inducing Mcl-1 upregulation [87]. CD38 also binds to α4β1 integrin and enhances the apoptosis resistance of CLL cells [88]. It is thought that cell surface receptor internalization triggers intracellular cell signaling [89]. Here, the surface levels of NGAL-R fell in CLL cells treated with NGAL, while NGAL did not influence the expression level of cell surface CD38, CD29/β1, and CD44 (Figure S5), suggesting that NGAL-R was not bound to any of these three receptors. In future work, it will be important to understand how NGAL/NGAL-R recruits and activates an Src kinase. In summary, the results of the present study support the signaling model presented in Figure 8. This model indicates that NGAL (by binding to NGAL-R) promotes the resistance to apoptosis of primary CLL cells through the sequential activation of an Src kinase, STAT3 and Mcl-1, leading to the further inhibition of both ΔΨ_m_ disruption, caspase-3 activation and DNA fragmentation. The ability of leukemic cells to release NGAL suggests the existence of an autocrine stimulation loop for CLL cell survival.

## 4. Methods

### 4.1. Ethics Statement

In line with the ethical tenets of the Declaration of Helsinki, all the patients provided their written and informed consent to participation in the study. The study protocol was approved by the ethical committe on human experimentation at Pitié-Salpêtrière Hospital on 21 May 2014 (CPPIDF6, Paris, France). Specific written, informed consent was not required for experiments on the control blood samples. 

### 4.2. Patients, Serum and Cell Separation

Peripheral blood was collected from 85 patients diagnosed with CLL according to standard clinical criteria and the International Workshop on CLL (IWCLL) criteria [3] including lymphocyte morphology, Binet stage, *IGHV* mutation status. Deletions of 17p13, 11q22, 13q14 and trisomy 12 were detected using fluorescence in situ hybridization (FISH) with the Metasystems XL DLEU/LAMP/12cen and XL ATM/TP53 Multi-Color Probe Kits (MetaSystems, Compiègne, France). The main exclusion criteria were as follows: neoplastic disease other than CLL, and current symptoms of ischemia, diabetes, hepatic fibrosis, renal disease or lung disease. Responses were evaluated using a physical examination and laboratory results according to the IWCLL response criteria. Control blood samples from healthy, anonymous donors were purchased from the French Blood Establishment (Etablissement Français du Sang, Paris, France). The biological and clinical characteristics of CLL patients are listed in Table 1.

The serum was immediately separated, aliquoted, and frozen at −80 °C pending further analyses. Peripheral blood mononuclear cells (PBMCs) were isolated from blood using Ficoll-Hypaque density gradient (1.077 g/mL) centrifugation. Normal B cells were purified from healthy donors’ PBMCs by negative selection with magnetic microbeads coupled to anti-CD3 and anti-CD14 monoclonal antibodies (Miltenyi Biotech, Paris, France) so as to avoid possible B cell activation. Cells were immunostained, as previously described [49], and analyzed by flow cytometry. More than 90% of CLL PBMCs were CD19^+^CD5^+^ and more than 90% of purified B cells were CD19^+^. Freshly isolated cells were used immediately in in vitro culture assays. Cell pellets were frozen at −80 °C until RNA or protein extraction, and analysis.

### 4.3. Cell Culture Conditions

Freshly isolated PBMCs (10^6^/mL) were cultured in RPMI 1640 medium (Life Technologies, Villebon-Sur-Yvette, France) supplemented with 2 mM L-glutamine, 1 mM sodium pyruvate, and 40 μg/mL gentamycin, in a 5% CO_2_ humidified atmosphere at 37 °C. To avoid possible interference between calf serum NGAL and exogenous NGAL, we used serum-free media in our tests. The cells were then treated with recombinant human (rh) NGAL (100 nM; Sigma, Saint Louis, MO, USA; R&D Systems, Abingdon, UK), etoposide (1 μM; Sigma) or rhIFN-γ (1000 U/mL; R&D Systems) for various periods of time. In some experiments, cells were pretreated with AG490 (Jak2 inhibitor, 10 μM; Calbiochem, Darmsdat, Germany), PP2 (Src inhibitor, 10 μM; Calbiochem), Stattic (STAT3 inhibitor, 5 μM; Calbiochem), anti-NGAL-R antibodies (20 μg/mL; rabbit Ig; Biorbyt, Cambridge, UK) or rabbit Ig (20 μg/mL; Sigma) for 15 min prior to the addition of NGAL or IFN-γ. In negative control experiments, cells were treated with the same volume of PBS or DMSO alone. The supernatants were collected and stored at −80 °C until titration. After incubation, the cells were collected, washed once, and then used for flow cytometry, DNA fragmentation, mitochondrial membrane permeability and caspase-3 activity assays, RT-PCR and Western blot analyses.

### 4.4. ELISAs

Concentrations of free NGAL (monomer and dimer) and NGAL complexed to proMMP-9 (CPX) in serum and cell culture supernatants (collected after 48 h) were determined using commercial ELISA kits (R&D Systems) according to the manufacturer’s instructions. The sensitivity was 12 pg/mL for NGAL and 13 pg for CPX. The coefficients of variation of the intra- and inter-assay values were respectively 3.6–4.4% and 5.6–7.9% for NGAL, 2.3–4.1% and 5.1–7.6% for CPX. Large variations in NGAL values exist within the healthy population (median values ranging from 18.9–153 ng/mL) [90,91,92].

### 4.5. Flow Cytometry

Intact cells were directly immunostained as previously described [49] with phycoerythrin (PE)-conjugated anti-CD19 (clone 4G7, mIgG1; Santa-Cruz, Heidelberg, Germany), anti-CD5-PE (clone 205919, mIgG1; R&D Systems), anti-CD14-PE (clone 134620, mIgG1; R&D Systems), anti-CD3-PE (clone SK7, mIgG1; BD Biosciences, Le Pont de Claix, France), fluorescein isothiocyanate (FITC)-conjugated anti-CD38 (clone HB7, mouse IgG1; BD Biosciences), and anti-NGAL-R (Slc22A17)-FITC (clone 16315, rabbit IgG; CliniSciences, Nanterre, France). Isotypes included FITC-mIgG1, PE-mIgG1 and FITC-rabbit IgG (Santa-Cruz). Intracellular active caspase-3 was detected using a specific FITC-conjugated rabbit IgG (C92-605, rabbit Ig; BD Biosciences) in cells permeabilized with the BD Cytofix/Cytoperm kit (BD Biosciences); the negative control was FITC-rabbit IgG. The balance between cell death and survival was assessed using the annexin V-FITC/propidium iodide (PI) cell death detection kit (Beckman-Coulter, Les Ullis, France). Mitochondrial transmembrane potential (ΔΨ_m_) was analyzed using the fluorescent dye cell permeant tetramethyl rhodamine ethyl ester (TMRE, 125 nM) (Life Technologies). Stained cells were analyzed with a Coulter Epics XL flow (Beckman-Coulter) or a FACSCanto II flow (BD Biosciences) cytometer. Data were analyzed using LYSYS (Beckman-Coulter) or FloJo (BD Biosciences) software.

### 4.6. DNA Fragmentation Assay

DNA fragmentation was assessed as described previously [34]. The DNA fragments were electrophoretically separated in 1.8% agarose gels containing ethidium bromide, and the gel bands were analyzed using a Quantum ST4 system (Vilber Lourmat, Marne La Vallée, France).

### 4.7. Real Time PCR Assays

RNA extraction from treated cells and cDNA synthesis were performed as described previously [49]. The cDNAs coding for human NGAL, SLC22A17/NGAL-R, Mcl-1, STAT3, and β2-microglobulin were amplified in PCRs, using primers synthesized by Sigma-Proligo and Eurofins Genomics, according to the published sequences [49,93,94,95,96]. The PCR products were visualized by electrophoresis in a 1.8% agarose gel containing 0.2 μg/mL ethidium bromide. The bands were imaged in a Quantum ST4 system (Vilber Lourmat, Marne La Vallée, France) and quantified using ImageJ64 software (NIH, Bethesda, MD, USA).

### 4.8. Immunoblotting

Cells were lysed in M-PER buffer (Pierce Biotechnology, Rockford, IL, USA) supplemented with protease and phosphatase inhibitor cocktails (Sigma). Total cell extracts were separated using SDS-PAGE in 10% gels, transferred to nitrocellulose membranes, and blotted as described previously [97]. The primary antibodies included anti-NGAL (clone AF1757, goat IgG, specific for the dimeric and monomeric forms; R&D Systems), anti-MMP-9 (clone EP1254, rabbit Ig, specific for CPX; Abcam, Paris, France), anti-NGAL-R/Slc22A17 (clone 75010, rabbit Ig; Biorbyt), and antibodies against Mcl-1 (clone S-19, rabbit IgG; Santa-Cruz), Bcl-2 (clone 100, mouse IgG1; Santa-Cruz), STAT3 (clone C20, rabbit IgG; Santa-Cruz), phospho-Tyr^705^-STAT3 (clone 710093, rabbit Ig; Thermo Fisher Scientific, Waltham, MA, USA), STAT1 (clone -23, rabbit IgG; Santa-Cruz) and actin (clone C4, mouse IgG1; ICN Biomedicals, Solon, OH, USA). Immunoreactive proteins were detected using horseradish peroxidase-conjugated secondary antibodies and visualized with the Pierce ECL Western blotting substrate system or the SuperSignal West Femto Maximum Sensitivity Substrate system (both from Thermo Fisher Scientific). Immunoblot images were acquired in an MF-ChemiBIS 4.2 imager (DNR Bio-Imaging Systems Ltd., Neve Yamin, Israel) and quantified using ImageJ64 software.

### 4.9. Statistics

All statistical analyses were performed using GraphPad Prism software (version 7.0, GraphPad Software, La Jolla, CA, USA). Data were expressed as the median or mean ± standard error of the mean (SEM). Groups were compared using Mann–Whitney tests or unpaired or paired Student’s t-tests. Correlations between variables were tested by calculating Spearman’s coefficient. For greater stringency, all tests were two-tailed. Significance levels were defined as * *p* < 0.05; ** *p* < 0.01; and *** *p* < 0.001.

## 5. Conclusions

The main treatments currently prescribed in an indication of CLL are FCR, rituximab, ibrutinib and venetoclax [98]. Unfortunately, these treatments often lead to adverse drug reactions or favor drug resistance mutations [99,100]. A multitude of different combination treatments containing these novel agents are currently being investigated [100,101]. Furthermore, expected goals in CLL research are the identification of new targets sustaining the survival of malignant B cells and the subsequent development of therapeutic agents that block the expression or the activity of these targets. The data presented here indicate that the elevated expression of NGAL and NGAL-R in untreated CLL patients is an intrinsic feature of the disease. Although the relevance of our observations remains to be established in vivo, the contribution of NGAL in the resistance to apoptosis of leukemic cells, through STAT3/Mcl-1 signaling, appears plausible. Hence, preventing NGAL from exerting its harmful action in vivo might be of value for improving CLL therapy. The inhibition of STAT3 or Mcl-1 could provide a therapeutic benefit by disrupting the NGAL-dependent signaling pathway that favors CLL cell survival. To our knowledge, direct STAT3 inhibitors of clinical grade are not yet available [102]. The development of potent small-molecule inhibitors specific for Mcl-1 have been reported in the literature [103] and, currently, six phase 1 clinical trials are underway for hematological malignancies, among other cancers [104]. Additionally, the ability of NGAL-R antibodies to promote CLL cell death might provide a new experimental tool for apoptosis-based therapeutic strategies in CLL.

## Figures and Tables

**Table 1:** Clinical characteristics of chronic lymphocytic leukemia (CLL) patients.

Patient	Sex/Age	Stage	*IGHV* Mutation	FISH	Karyotype	Therapy
1	M/71	A	M	Trisomy 12	1 abnormality	None
2	M/63	A	ND	13q-	Complex	None
3	M/90	A	M	ND	ND	None
4	M/88	A	M	13q-	Normal	None
5	M/76	A	ND	13q-	Normal	None
6	M/87	C	M	17p-, 13q-	Normal	None
7	M/66	B	M	13q-	2 abnormalities	None
8	M/71	A	ND	13q-	Normal	None
9	M/71	A	M	11q-, 13q-	1 abnormality	None
10	F/69	B	ND	13q-	Normal	None
11	F/74	A	UM	13q-	Normal	None
12	F/74	A	M	Normal	Normal	None
13	F/78	A	M	13q-	Normal	None
14	M/73	A	M	Normal	Normal	None
15	F/68	A	ND	13q-	1 abnormality	None
16	F/62	B	UM	13q-	Normal	None
17	F/69	A	M	13q-	Normal	None
18	F/87	A	ND	13q-	Normal	None
19	F/83	A	ND	Normal	Normal	None
20	M/86	A	ND	Normal	Normal	None
21	M/86	A	M	Trisomy 12	Complex	None
22	M/82	A	ND	11q-, 13q-	Normal	None
23	F/77	A	UM	13q-	Failure	None
24	M/74	A	ND	13q-	2 abnormalities	None
25	M/86	A	M	13q-	ND	None
26	F/76	A	UM	11q-, 13q-	Normal	None
27	F/88	A	ND	ND	ND	None
28	F/64	A	M	13q-	Normal	None
29	M/70	A	ND	13q-	Normal	None
30	M/75	A	M	13q-	Normal	None
31	F/67	A	ND	13q-	Normal	None
32	F/74	A	ND	13q-	1 abnormality	None
33	M/76	A	ND	13q-	Normal	None
34	M/68	B	ND	11q-	Complex	None
35	F/85	A	M	13q-	Normal	None
36	F/70	A	ND	13q-	Normal	None
37	F/52	A	ND	13q-	Normal	None
38	F/79	A	ND	13q-	Normal	None
39	F/54	B	ND	Trisomy 12	1 abnormality	None
40	M/76	A	M	13q-	Normal	None
41	F/57	A	ND	13q-	Normal	None
42	M/72	A	M	13q-	ND	None
43	F/84	A	ND	Normal	Normal	None
44	F/80	B	ND	11q-	1 abnormality	None
45	M/62	A	ND	Normal	Normal	None
46	M/75	B	UM	Trisomy 12	1 abnormality	None
47	F/73	A	ND	13q-	Failure	None
48	M/59	A	ND	ND	ND	None
49	F/75	A	ND	13q-	Normal	None
50	M/61	B	ND	13q-, Trisomy 12	Complex	None
51	F/82	A	ND	13q-	Normal	None
52	F/53	A	UM	13q-	2 abnormalities	None
53	M/71	A	UM	11q-, 13q-	ND	None
54	F/96	A	UM	Trisomy 12	1 abnormality	None
55	M/72	A	ND	Normal	Normal	None
56	F/85	A	ND	Normal	2 abnormalities	None
57	F/69	A	M	Normal	Normal	None
58	F/51	B	UM	11q-, 13q-	1 abnormality	None
59	M/63	B	UM	13q-	Normal	None
60	M/70	B	UM	11q-, 13q-	2 abnormalities	None
61	F/80	B	ND	11q-	1 abnormality	Ibrutinib/relapse
62	M/62	B	UM	11q-, 13q-	1 abnormality	FCR/relapse
63	F/78	A	M	13q-	1 abnormality	RCb/relapse
64	M/54	B	UM	13q-	2 abnormalities	Ibrutinib/remission
65	F/78	C	UM	17p-, 13q-, Trisomy 12	Complex	Ibrutinib/remission
66	M/76	B	ND	17p-, 13q-	2 abnormalities	Ibrutinib/relapse
67	M/80	B	UM	17p-, 13q-	Complex	Ibrutinib/relapse
68	M/54	C	UM	17p-, 11q-	Complex	Ibrutinib/remission
69	F/72	B	UM	17p-, Trisomy 12	Complex	Ibrutinib/remission
70	M/92	A	ND	ND	ND	Cb/relapse
71	M/66	C	UM	13q-, Trisomy 12	Complex	Ibrutinib/remission
72	M/70	A	M&UM	11q-	Failure	FCR/relapse
73	F/77	A	M	Normal	ND	FC/relapse
74	M/88	C	UM	17p-, Trisomy 12	Normal	RCb/remission
75	M/82	C	UM	11q-, 13q-	Complex	BR/relapse
76	M/79	B	UM	Normal	Normal	BR/remission
77	M/64	A	UM	Trisomy 12	1 abnormality	FCR/cyclosporine/remission
78	F/75	B	M	13q-, Trisomy 12	1 abnormality	FCR/remission
79	F/84	B	ND	Normal	Complex	Ibrutinib/remission
80	F/64	B	UM	13q-	Normal	FCR/remission
81	M/69	B	ND	11q-	Complex	FCR x4/remission
82	M/75	B	M	Trisomy 12	1 abnormality	BR/remission
83	M/74	C	M	11q-, 13q-	ND	MiniCHOP/remission
84	M/81	C	M	11q-, 13q-	ND	Alemtuzumab/relapse
85	M/75	B	UM	17p-, 13q-	2 abnormalities	FCR x2/relapse

Unmutated (UM); mutated (M); not done (ND). Fluorescent in situ hybridization (FISH) detecting 13q14 deletion (13q-), 11q22 deletion (11q-), 17p13 deletion (17p-) and trisomy 12. Chromosomal abnormalities are defined by conventional karyotyping (complex karyotype for ≥3 chromosomal abnormalities). Fludarabine, cyclophosphamide and rituximab (FCR). Rituximab and chlorambucil (RCb). Chlorambucil (Cb). Fludarabine and cyclophosphamide (FC). Bendamustine and rituximab (BR). Cyclophosphamide, vincristine and prednisone (MiniCHOP).

**Table 2:** Lymphocyte and neutrophil counts of patients P16 and P46.

**Patient P16/Months**	**Lymphocyte Count (G/L)**	**Neutrophil Count (G/L)**
0	159.6	12.55
5	179.86	26
7	189.38	12.91
9	196.14	2.13
11	239.94	4.0
12	248.5	5.29
19 (treated)	0.26	0.80
27 (treated)	0.59	2.28
Correlation with:	*p*-value	*p*-value
serum NGAL	0.0011	0.6191
serum CPX	0.0011	0.6191
**Patient P46/Months**	**Lymphocyte Count (G/L)**	**Neutrophil Count (G/L)**
0	146	1.5
1.5	184	3.9
4	219.5	2.3
10 (treated)	1.11	4.39
Correlation with:	*p*-value	*p*-value
serum NGAL	0.0833	0.7500
serum CPX	0.3333	0.3333

*p*-Value represents statistical significance. Correlations between variables (lymphocyte count, neutrophil count) and serum levels of NGAL and CPX were calculated using the Spearman’s test.

## Supplementary Materials

The following are available online at https://www.mdpi.com/2072-6694/12/8/2124/s1, **Figure S1.** The release of NGAL by CLL cells from untreated patients (n = 33) and normal isolated B cells (n = 9) after a 48 h culture time-course, were quantified by ELISA; *p-*value was calculated using a Mann-Whitney *U*-test. Representative Western blot of NGAL dimer released in the supernatant of cultured CLL cells from patient P36. The primary antibody used was anti-NGAL specific for the dimeric and monomeric forms (clone AF1757, goat IgG; R&D Systems). **Figure S2.** Representative Western blot (non reducing conditions) results for recombinant NGAL proteins sold by Sigma (dimers) and R&D systems (monomers); a control includes endogenous NGAL protein (dimers and monomers) in a CLL cell sample (P28). **Figure S3.** Ibrutinib affects neither NGAL-mediated CLL survival nor the surface expression of NGAL-R. (**A)** CLL cells (P56) were cultured for 36 h in the presence or absence of NGAL monomers (100 nM) or IFN-γ (1000 U/mL) after a 15 min pretreatment with 5 μM ibrutinib. Detection of apoptotic cells after annexin-V-FITC staining and flow cytometry. The percentage of annexin-V-positive cells is shown. (**B)** CLL cells (P56) were cultured for 36 h in the presence or absence of 5 μM ibrutinib after which cells were stained with rabbit IgG-FITC or NGAL-R-FITC and then examined by flow cytometry. **Figure S4.** NGAL does not affect STAT1 expression in CLL cells. CLL cells were cultured for 24 h in the presence or absence of NGAL dimers or monomers (100 nM), IFN-γ (1000 U/mL) or etoposide (1 μM); after which lysates were Western blotted (reducing conditions) with antibodies against STAT1 and actin. Three representative experiments are shown. Data (n = 3) are expressed as the ratio between the analyte protein and actin, and presented as mean ± SEM. Statistical relevance was assessed with the unpaired *t*-test. As control, IFN-γ upregulates the level of STAT1 protein. **Figure S5.** NGAL does not affect the surface expression of CD38, β1 and CD44 antigens in CLL cells. Representative cytograms of CLL cells (P28) cultured for 24 h in the presence or absence of NGAL dimers or monomers (20 nM); cells were stained with isotypes (negative controls; rabbit IgG-FITC or mIgG1-FITC, grey peaks) or anti-NGAL-R-FITC, anti-CD38-FITC, anti-β1-FITC or anti-CD44-FITC (white peaks) and then examined by flow cytometry. **Figure S6.** Whole blots relative to the Western Blotting analyses. **Table S1.** Correlations between serum levels of NGAL (free and complexed) and clinical characteristics of untreated CLL patients. *p*-value represents statistical significance. Correlations between variables (age, lymphocyte count, neutrophil count, CD38) and serum levels of NGAL and CPX were calculated using the Spearman’s test. Comparisons between groups (males and females, Binet stage A and stage B/C, *IGHV* UM and *IGHV* M, 11q- positive and 11q- negative, 13q- positive and 13q- negative, and trisomy 12 positive and trisomy 12 negative) were calculated using the Mann-Whitney *U*-test. The cohort included one untreated patient (P6) with 17p13 deletion.
