# Supplementary material for: Relation of Neutrophil Gelatinase-Associated Lipocalin Overexpression to the Resistance to Apoptosis of Tumor B Cells in Chronic Lymphocytic Leukemia
Source: Cancers (Basel). 2020 Jul 31;12(8):2124. doi: 10.3390/cancers12082124 (PMC7465759; doi:10.3390/cancers12082124)
Supplement: Supplementary file 1 [file cancers-12-02124-s001.pdf]

cancers

MDPI

Supplementary Materials

Relation of Neutrophil Gelatinase-Associated Lipocalin Overexpression to the Resistance to Apoptosis of Tumor B Cells in Chronic Lymphocytic Leukemia

Brigitte Bauvois, Elodie Pramil, Ludovic Jondreville, Elise Chapiro, Claire Quiney, Karim Maloum, Santos A. Susin and Florence Nguyen-Khac

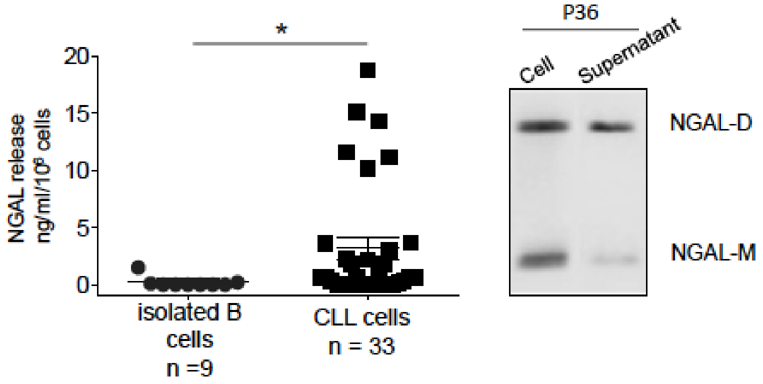

Figure S1. The release of NGAL by CLL cells from untreated patients (n = 33) and normal isolated B cells (n = 9) after a 48 h culture time-course, were quantified by ELISA; *P* value was calculated using a Mann-Whitney *U*-test. Representative Western blot of NGAL dimer released in the supernatant of cultured CLL cells from patient P36. The primary antibody used was anti-NGAL specific for the dimeric and monomeric forms (clone AF1757, goat IgG; R&D Systems).

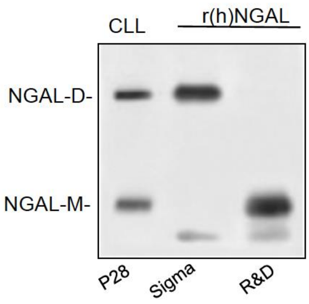

Figure S2. Representative Western blot (non reducing conditions) results for recombinant NGAL proteins sold by Sigma (dimers) and R&D systems (monomers); a control includes endogenous NGAL protein (dimers and monomers) in a CLL cell sample (P28).

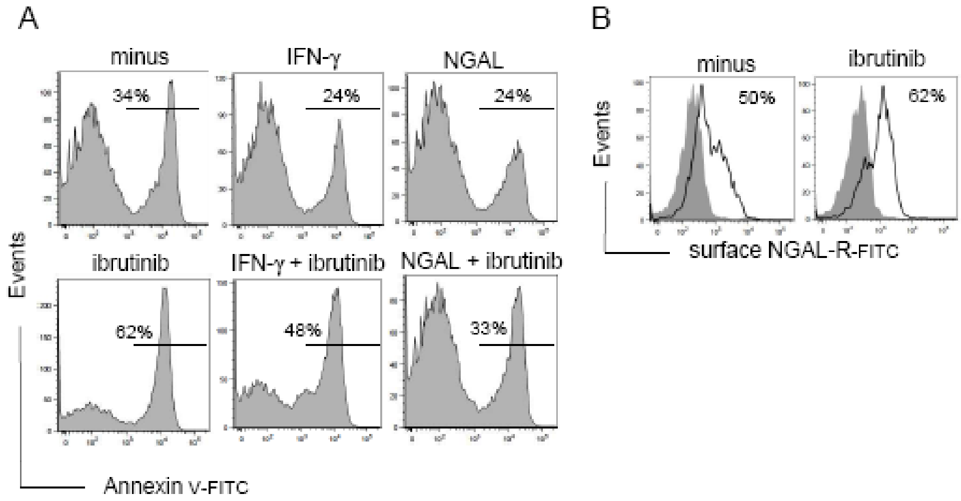

Figure S3. Ibrutinib affects neither NGAL-mediated CLL survival nor the surface expression of NGAL-R. (**A**) CLL cells (P56) were cultured for 36 h in the presence or absence of NGAL monomers (100 nM) or IFN-γ (1000 U/mL) after a 15 min pretreatment with 5 μM ibrutinib. Detection of apoptotic cells after annexin-V-FITC staining and flow cytometry. The percentage of annexin-V-positive cells is shown. (**B**) CLL cells (P56) were cultured for 36 h in the presence or absence of 5 μM ibrutinib after which cells were stained with rabbit IgG-FITC or NGAL-R-FITC and then examined by flow cytometry.

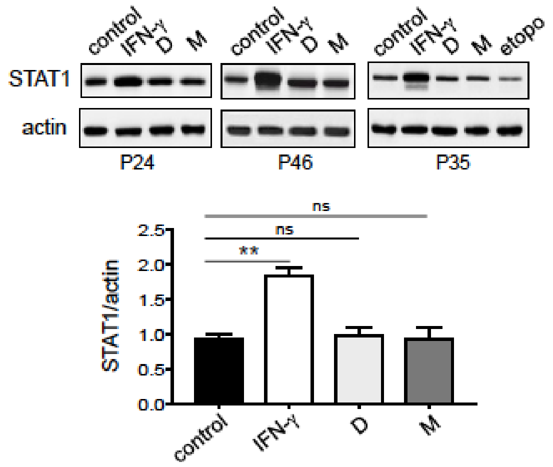

Figure S4. NGAL does not affect STAT1 expression in CLL cells. CLL cells were cultured for 24 h in the presence or absence of NGAL dimers or monomers (100 nM), IFN-γ (1000 U/mL) or etoposide (1 μM); after which lysates were Western blotted (reducing conditions) with antibodies against STAT1 and actin. Three representative experiments are shown. Data (n = 3) are expressed as the ratio between the analyte protein and actin, and presented as mean ± SEM. Statistical relevance was assessed with the unpaired *t*-test. As control, IFN-γ upregulates the level of STAT1 protein.

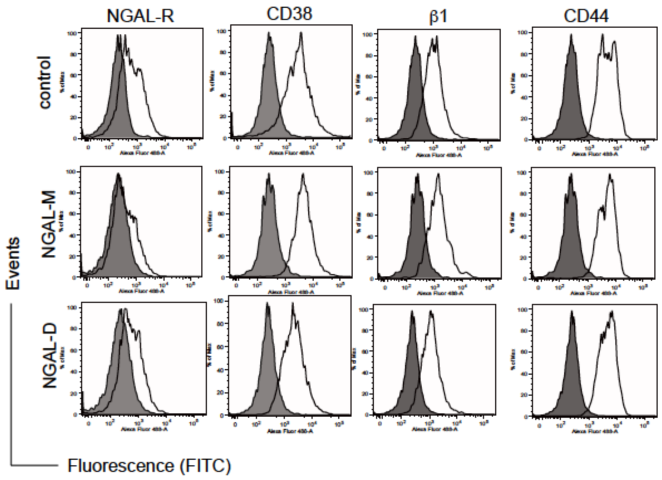

Figure S5. NGAL does not affect the surface expression of CD38, β1 and CD44 antigens in CLL cells. Representative cytograms of CLL cells (P28) cultured for 24 h in the presence or absence of NGAL dimers or monomers (20 nM); cells were stained with isotypes (negative controls; rabbit IgG-FITC or mIgG1-FITC, grey peaks) or anti-NGAL-R-FITC, anti-CD38-FITC, anti-β1-FITC or anti-CD44-FITC (white peaks) and then examined by flow cytometry.

(A) For Figure 1E:

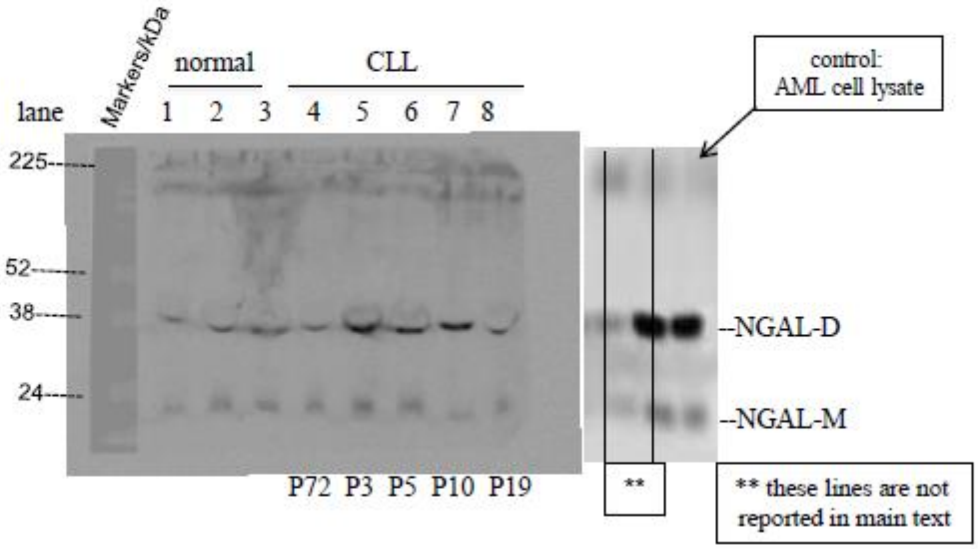

lane	1 : normal	2 : normal	3 : normal	4 : CLL P72	5 : CLL P3	6 : CLL P5	7 : CLL P10	8 : CLL P19
monomer/dimer, % (ratio M/D x 100)	17	20	17	24	17	15	12	16

(B) For Figure 2B:

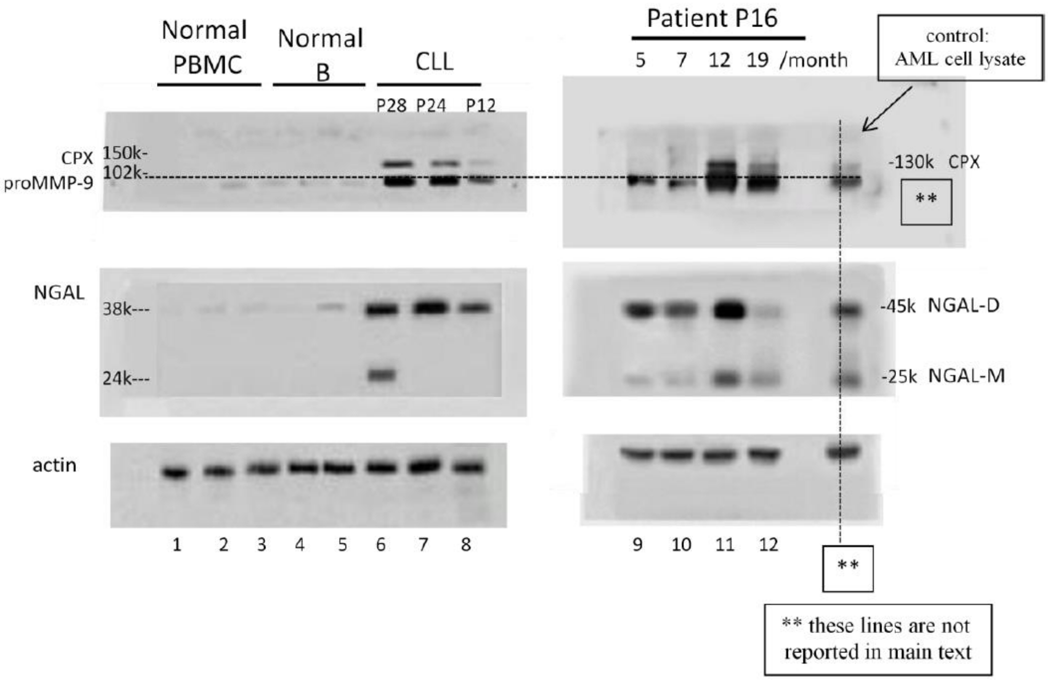

lane	NGAL-D/actin	NGAL-M/actin	CPX/actin
1 : Normal PBMC	0.03	-	-
2 : Normal PBMC	0.10	-	-
3 : Normal PBMC	0.09	-	-
4 : Normal isolated B	0.02	-	-
5 : Normal isolated B	0.11	-	-
6 : CLL P28	1.12	0.69	1.36
7 : CLL P24	1.02	-	0.72
8 : CLL P12	0.65	-	0.21
9 : CLL P16, 5 months	0.84	0.12	0.22
10 : CLL P16, 7 months	0.76	0.18	0.23
11 : CLL P16, 12 months	1.41	0.70	1.36
12 : CLL P16, 19 months/treated	0.13	0.20	0.63

(C) For Figure 3F

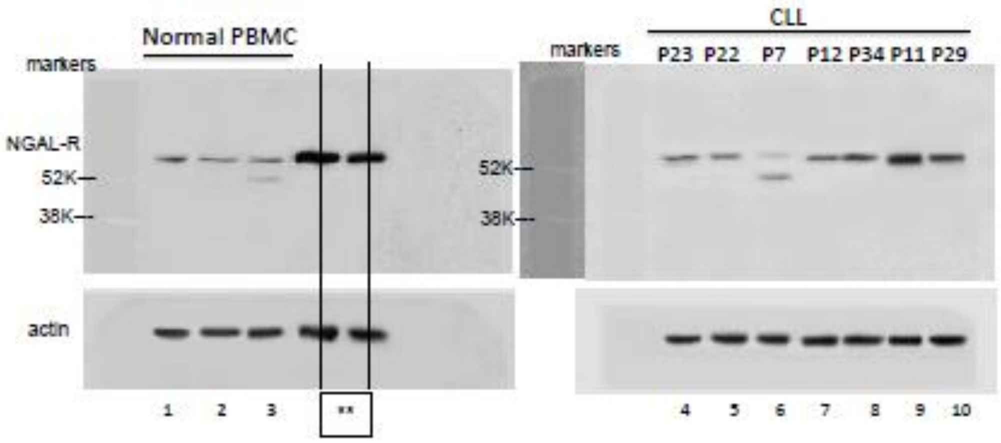

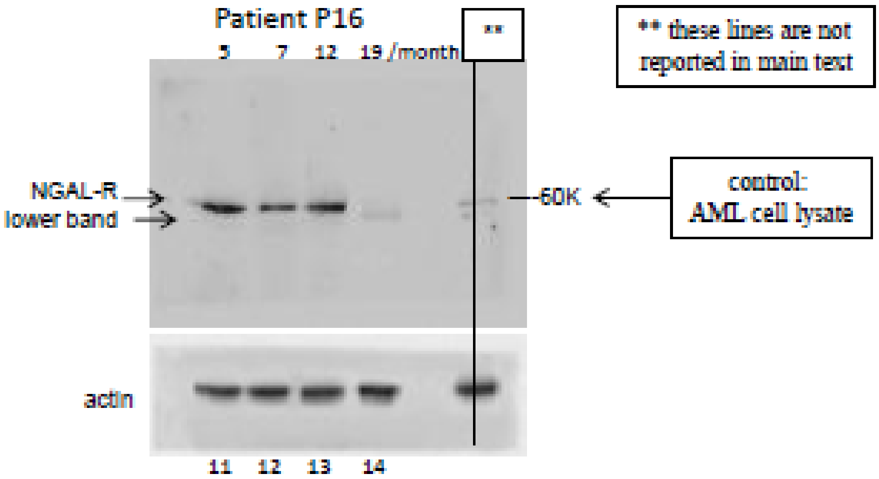

The blot of Patient P16 is the one previously used in Figure 2B.

**lane**	**1: normal PBMC**	**2: normal PBMC**	**3: Normal PBMC**	**4: CLL P23**	**5: CLL P22**	**6: CLL P7**	**7: CLL P12**	**8: CLL P34**	**9: CLL P11**	**10: CLL P29**
NGAL-R 60 kDa /actin	0.33	0.28	0.28	0.56	0.32	0.10	0.46	0.59	1.22	0.71
lower band /actin			0.15			0.29				
lane	11: CLL P16 5 months	12: CLLP16 7 months	13: CLL P16 12 months	14: CLL P16 19 months/treated						
NGAL-R 60 kDa /actin	1.46	1.04	1.09	-						
lower band /actin				0.28						

(D) For Figure 6D

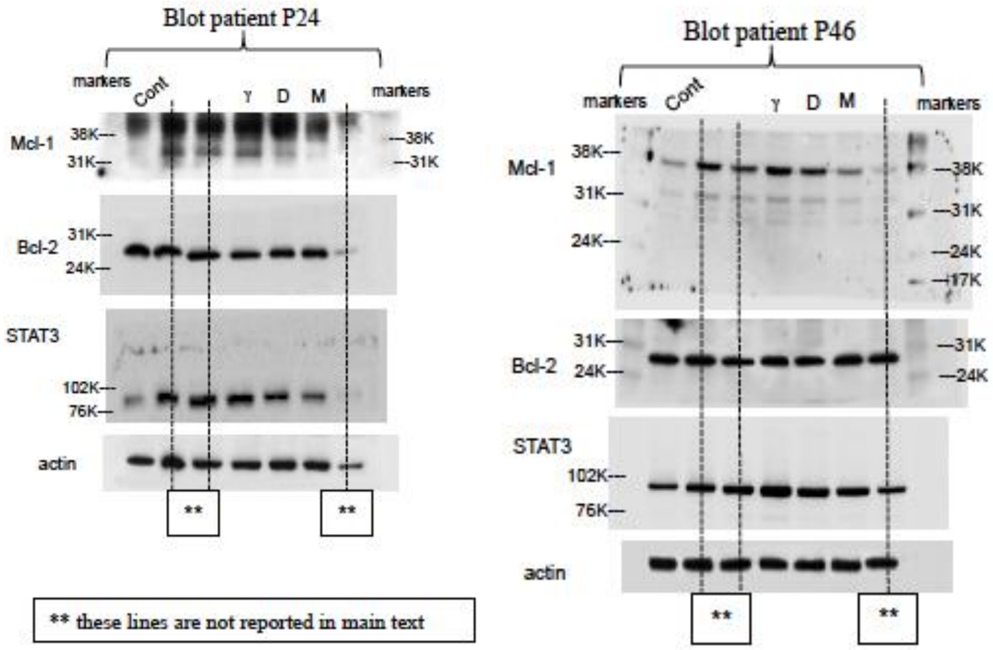

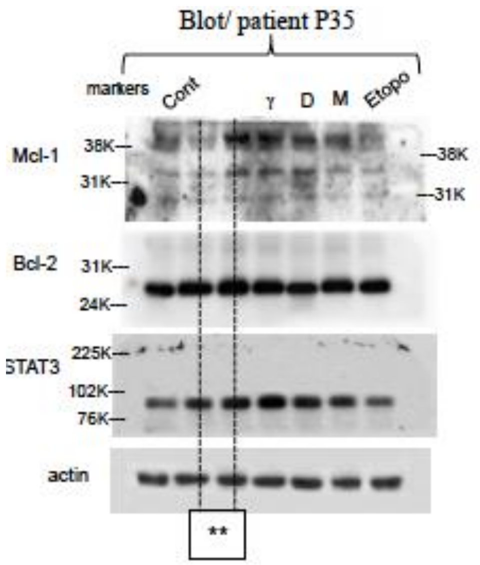

Blot patient	Control	IFN-γ	NGAL-D	NGAL-M	Etoposide
P24 Mcl-1/actin	1.0	2.0	1.5	1.2	-
P24 Bcl-2/actin	1.0	1.2	0.9	1.0	-
P24 STAT3/actin	1.0	2.7	2.1	1.7	-
P46 Mcl-1/actin	1.0	5.1	2.8	1.9	-
P46 Bcl-2/actin	1.0	0.9	0.8	1.0	-
P46 STAT3/actin	1.0	2.7	1.5	1.5	-
P35 Mcl-1/actin	1.0	2.9	1.6	1.5	0.3
P35 Bcl-2/actin	1.0	1.2	1.0	1.1	1.0
P35 STAT3/actin	1.0	2.2	1.6	1.5	1.0

Control ratios were set to 1.0 to allow comparison between groups.

(E) For Figure 7C:

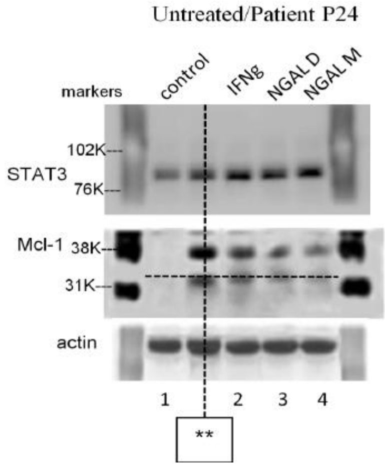

** this line is not reported in main text

lane	1: control	2: IFNg	3: NGAL D	4: NGAL M
Ratio STAT3/actin	1.0	2.4	2.8	2.5
Ratio Mcl-1/actin	1.0	7.1	5.0	4.2

Control ratios were set to 1.0 to allow comparison between groups (control, Stattic, PP2).

(F) For Figure 7C:

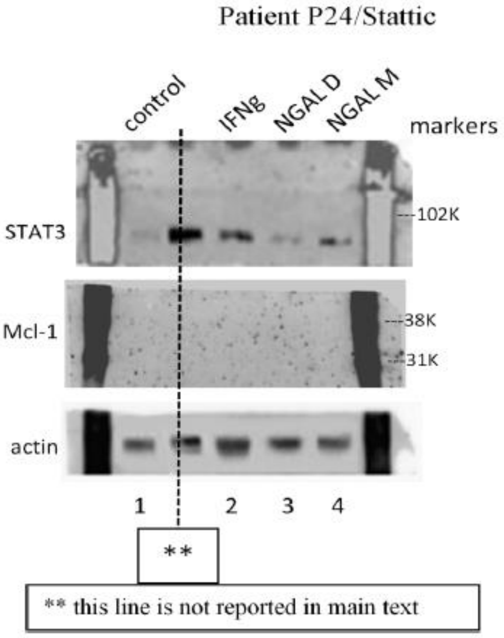

lane	1: + stattic	2: IFNg + stattic	3: NGAL D + stattic	4: NGAL M + stattic
Ratio STAT3/actin	1.0	1.9	1.1	1.3
Ratio Mcl-1/actin	-	-	-	-

(G) For Figure 7C:

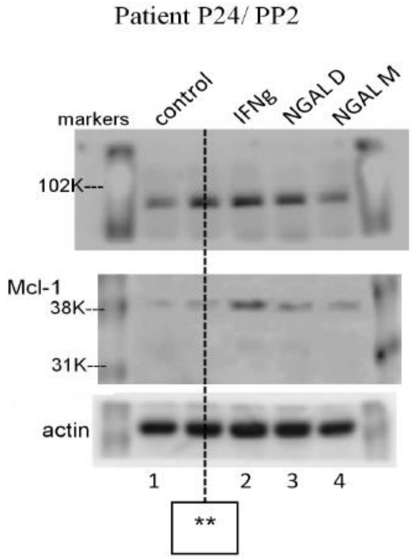

lane	1: +PP2	2: IFNg +PP2	3: NGAL D + PP2	4: NGAL M + PP2
Ratio STAT3/actin	1.0	1.9	1.6	1.2
Ratio Mcl-1/actin	1.0	4.2	1.3	1.6

(H) For Figure 7E :

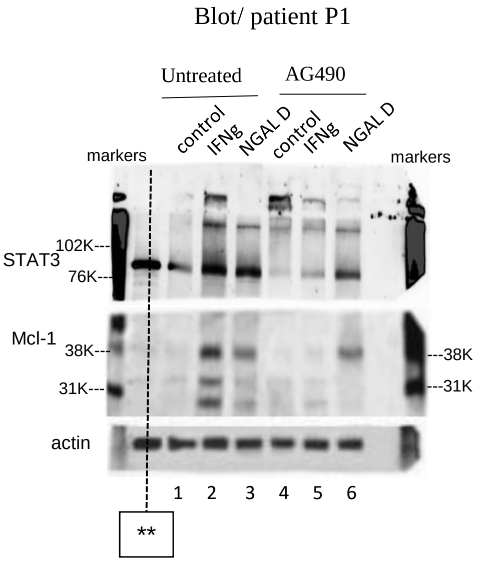

Patient P1 lane	1 : control	2 : IFNg	3 : NGAL D	4 : Control + AG490	5 : IFNg + AG490	6 : NGAL D + AG490
Ratio STAT3/actin	1.0	2.5	2.3	1.0	1.2	2.6
Ratio Mcl-1/actin	1.0	7.6	5.9	1.0	1.0	5.0

(I) For Figure 7E :

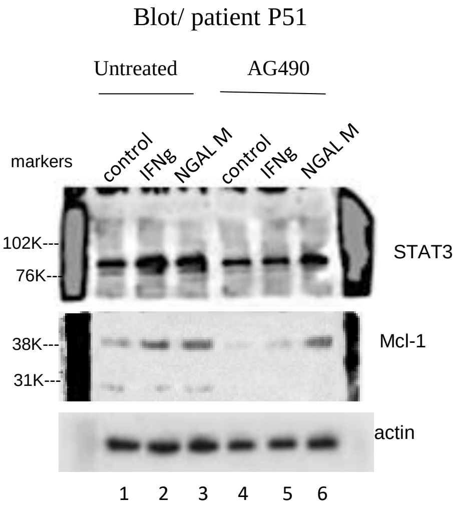

Patient P51 lane	1 : control	2 : IFNg	3 : NGAL D	4 : Control + AG490	5 : IFNg + AG490	6 : NGAL D + AG490
Ratio STAT3/actin	1.0	1.9	1.6	1.0	1.1	1.5
Ratio Mcl-1/actin	1.0	2.2	2.3	1.0	1.3	2.4

Figure S6. Whole blots relative to the Western Blotting analyses.

Table S1. Correlations between serum levels of NGAL (free and complexed) and clinical characteristics of untreated CLL patients.

Clinical characteristic	Serum NGAL *p*- value	Serum CPX *p*-value
Age (n = 56)	0.10	0.92
Lymphocyte count (n = 49)	0.84	0.97
Neutrophil count (n = 49)	0.15	0.21
CD38 (n = 29)	0.23	0.32
Male (n = 25) *vs* female (n = 31)	0.78	0.36
Stage A (n = 46) *vs* B/C (n = 10)	0.82	0.57
IGHV : UM (n = 8) *vs* M (n = 17)	0.59	0.63
11q- positive (n = 6) *vs* 11q- negative (n = 47)	0.99	0.16
13q- positive (n = 38) *vs* 13q- negative (n = 15)	0.14	0.95
Trisomy 12 positive (n = 6) *vs* Trisomy 12 negative (n = 48)	0.30	0.09

p-value represents statistical significance. Correlations between variables (age, lymphocyte count, neutrophil count, CD38) and serum levels of NGAL and CPX were calculated using the Spearman's test. Comparisons between groups (males and females, Binet stage A and stage B/C, *IGHV* UM and *IGHV* M, 11q- positive and 11q- negative, 13q- positive and 13q- negative, and trisomy 12 positive and trisomy 12 negative) were calculated using the Mann-Whitney *U*-test. The cohort included one untreated patient (P6) with 17p13 deletion.
